# Adjunct clinical interventions that influence vaginal birth after cesarean rates: systematic review

**DOI:** 10.1186/s12884-018-2065-x

**Published:** 2018-11-21

**Authors:** Aireen Wingert, Cydney Johnson, Robin Featherstone, Meghan Sebastianski, Lisa Hartling, R. Douglas Wilson

**Affiliations:** 1 Alberta Research Centre for Health Evidence, Department of Pediatrics, University of Alberta, Edmonton, AB Canada; 2 Alberta Strategy for Patient-Oriented Research (SPOR) SUPPORT Unit Knowledge Translation Platform, University of Alberta, Edmonton, AB Canada; 3 Department of Obstetrics and Gynecology, Cumming School of Medicine, University of Calgary, 1403 – 29 Street NW, Calgary, AB T2N 2T9 Canada

**Keywords:** Vaginal birth after cesarean, Trial of labor after cesarean, Systematic review

## Abstract

**Background:**

Rates of cesarean deliveries have been increasing, and contributes to the rising number of elective cesarean deliveries in subsequent pregnancies with associated maternal and neonatal risks. Multiple guidelines recommend that women be offered a trial of labor after a cesarean (TOLAC). The objective of the study is to systematically review the literature on adjunct clinical interventions that influence vaginal birth after cesarean (VBAC) rates.

**Methods:**

We searched Ovid Medline, Ovid Embase, Wiley Cochrane Library, CINAHL via EBSCOhost; and Ovid PsycINFO. Additional studies were identified by searching for clinical trial records, conference proceedings and dissertations. Limits were applied for language (English and French) and year of publication (1985 to present). Two reviewers independently screened comparative studies (randomized or non-randomized controlled trials, and observational designs) according to a priori eligibility criteria: women with prior cesarean sections; any adjunct clinical intervention or exposure intended to increase the VBAC rate; any comparator; and, outcomes reporting changes in TOLAC or VBAC rates. One reviewer extracted data and a second reviewer verified for accuracy. Two reviewers independently conducted methodological quality assessments using the Mixed Methods Appraisal Tool (MMAT).

**Results:**

Twenty-three studies of overall moderate to good methodological quality examined adjunct clinical interventions affecting TOLAC and/or VBAC rates: system-level interventions (three studies), provider-level interventions (three studies), guidelines or information for providers (seven studies), provider characteristics (four studies), and patient-level interventions (six studies). Provider-level interventions (opinion leader education, laborist, and obstetrician second opinion for cesarean sections) and provider characteristics (midwifery antenatal care, physicians on night float call schedules, and deliveries by family physicians) were associated with increased rates of VBAC. Few studies employing heterogeneous designs, sample sizes, interventions and comparators limited confidence in the effects. Studies of system-level and patient-level interventions, and guidelines/information for providers reported mixed findings.

**Conclusions:**

Limited evidence indicates some provider-level interventions and provider characteristics may increase rates of attempted and successful TOLACs and/or VBACs, whereas other adjunct clinical interventions such as system-level interventions, patient-level interventions, and guidelines/information for healthcare providers show mixed findings.

**Electronic supplementary material:**

The online version of this article (10.1186/s12884-018-2065-x) contains supplementary material, which is available to authorized users.

## Background

A cesarean delivery is the most common surgery in Canada, with one of the main contributors being an elective cesarean delivery in subsequent pregnancies [1, 2]. Canadian cesarean delivery rates have increased from 18.7% in 1997 to 27.5% in 2014 [3] and continue to increase globally, [4] the result of an interplay of multiple factors including, but not limited to, shifting clinical environments, provider and patient preferences, and changing maternal demographics (e.g., obesity, chronic disease prevalence and advanced age) [5–9]. These factors can lead to higher-risk and more complex pregnancies and deliveries and an increased likelihood of a cesarean delivery [10, 11].

Depending on the etiology or indication, a cesarean delivery contributes to short- and long-term risks for both mother and infant [12, 13]. The Society of Obstetricians and Gynaecologists of Canada (SOGC) recommends that a trial of labor be offered to women with one previous transverse low-segment cesarean section [12]. A woman’s willingness to undergo a trial of labor after cesarean (TOLAC) may be influenced by a multitude of factors [14]. While a vaginal birth after a cesarean (VBAC) may be desired by some women, the patient-level benefits associated with a VBAC from avoiding major abdominal surgery and risk of complications in future pregnancies must be weighed against the potential for serious harms such as a failed TOLAC and subsequent maternal and neonatal morbidity including an unplanned repeat cesarean delivery [15]. For women with more than one previous cesarean delivery, a VBAC is likely to be successful, but with an estimated higher risk of uterine rupture (0.2 to 1.5% with a transverse uterine incision, 1.0 to 1.6% with a low-vertical uterine incision) [12]. This SOGC statement is consistent with recommendations from the American College of Obstetrics and Gynecology (ACOG) [16, 17].

This systematic review aimed to evaluate adjunct clinical interventions that could be directed at or used by patients, families, healthcare providers, and hospitals/health systems to influence the uptake and success of VBAC.

## Methods

This summation followed the standardized methods and guidelines for systematic reviews, [18, 19] and used an ‘a priori’ protocol (available from authors).

### Literature search

A research librarian searched the following databases in May 2017: Ovid Medline (1946-), Ovid Embase (1980-), Wiley Cochrane Library (inception-), CINAHL via EBSCOhost (1937-) and Ovid PsycINFO (1806-). Limits were applied for language (English and French) and publication year (1985). The search strategy used the Conference Proceedings Citation Indexes (Clarivate Analytics) and hand-searched meeting abstracts from the past 2 years from the following associations: The Society for Maternal-Fetal Medicine (SMFM), the Society of Obstetricians and Gynaecologists of Canada (SOGC), and the American Congress of Obstetricians and Gynecologists. Finally, we searched ClinicalTrials.gov and ProQuest Dissertations & Theses Global (1861-). Reference lists of relevant systematic reviews were reviewed for potentially eligible studies. The detailed search strategy is in Additional file 1: Appendix 1.

### Eligibility criteria

The study population was women who had a previous cesarean delivery including women with more than one prior cesarean delivery. Births attended by any healthcare provider (e.g., family physician, midwife, obstetrician/gynecologist) were eligible. Any intervention or exposure that was intended to effect a change in the VBAC rate among women with a prior cesarean delivery was eligible for inclusion. To be eligible, studies had to report on at least one of the outcomes of interest to the review: the primary outcome was change(s) in VBAC rates; secondary outcomes included TOLAC rates, or where reported, rates of successful VBAC among women undergoing a TOLAC. Studies that examined deliveries in any setting (e.g., hospitals, primary care centers, birthing units, home births) were eligible. All study designs (randomized [RCT] and non-randomized controlled trials [NRCT], and observational studies) with a comparison group were eligible for inclusion.

Studies were not considered eligible if: all women had three or more prior cesareans; multiple births of three or more fetuses were explicitly included; there was an absence of an exposure or intervention, or an inappropriate exposure/intervention was used (e.g., ethnicity, socioeconomic status, insurance status, physician traits, malpractice premiums); there was absence of a comparator, or an inappropriate comparator was used (e.g., no data for comparison groups in before-after study designs, women without a previous cesarean delivery); VBAC rates or change were not reported; or, they were not primary research (e.g., letter, editorial, commentary). Systematic reviews were not included; reference lists therein were screened for potentially relevant studies.

### Study selection

Two reviewers (CJ and AW) independently screened titles and abstracts using a priori eligibility criteria. Full texts of potentially relevant publications were retrieved and independently reviewed in duplicate for inclusion; disagreements were resolved through discussion or third-reviewer consultation.

### Data extraction

One reviewer extracted data and another verified data from each included study using a pre-specified and piloted form. Data were extracted for relevant study characteristics (design features), population (number of previous cesarean deliveries, parity), intervention, comparator, outcome (TOLAC rate [the number of women with a previous cesarean delivery who attempt a vaginal delivery] and VBAC rate [the number of women with a previous cesarean delivery who undergo a successful vaginal delivery]), funding source, and setting.

Intention-to-treat results were extracted from individual studies whenever possible. For dichotomous data on rates of TOLAC and VBAC, we reported counts or proportions, and sample size, by study arm. Results of statistical tests (e.g., *p*-values) or summary statistics (e.g., odds ratio [OR], risk ratio [RR], with confidence intervals [CI]) were extracted whenever these were reported within the studies.

### Assessment of methodological quality

Two reviewers independently assessed the methodological quality of included studies; disagreements were resolved via consensus. All studies were assessed using the Mixed Methods Appraisal Tool (MMAT [20]), a tool designed for systematic reviews that include multiple study designs.

### Data synthesis

Due to heterogeneity of interventions and comparators, pooling of data across studies for a meta-analysis was not appropriate; therefore, results were described narratively.

### Assessment of overall quality of evidence

Data from studies were not pooled for summary effect estimates; therefore, assessment of the quality of the body of evidence using the Grading of Recommendations Assessment, Development and Evaluation (GRADE [21]) was not conducted.

## Results

**Fig. 1:**
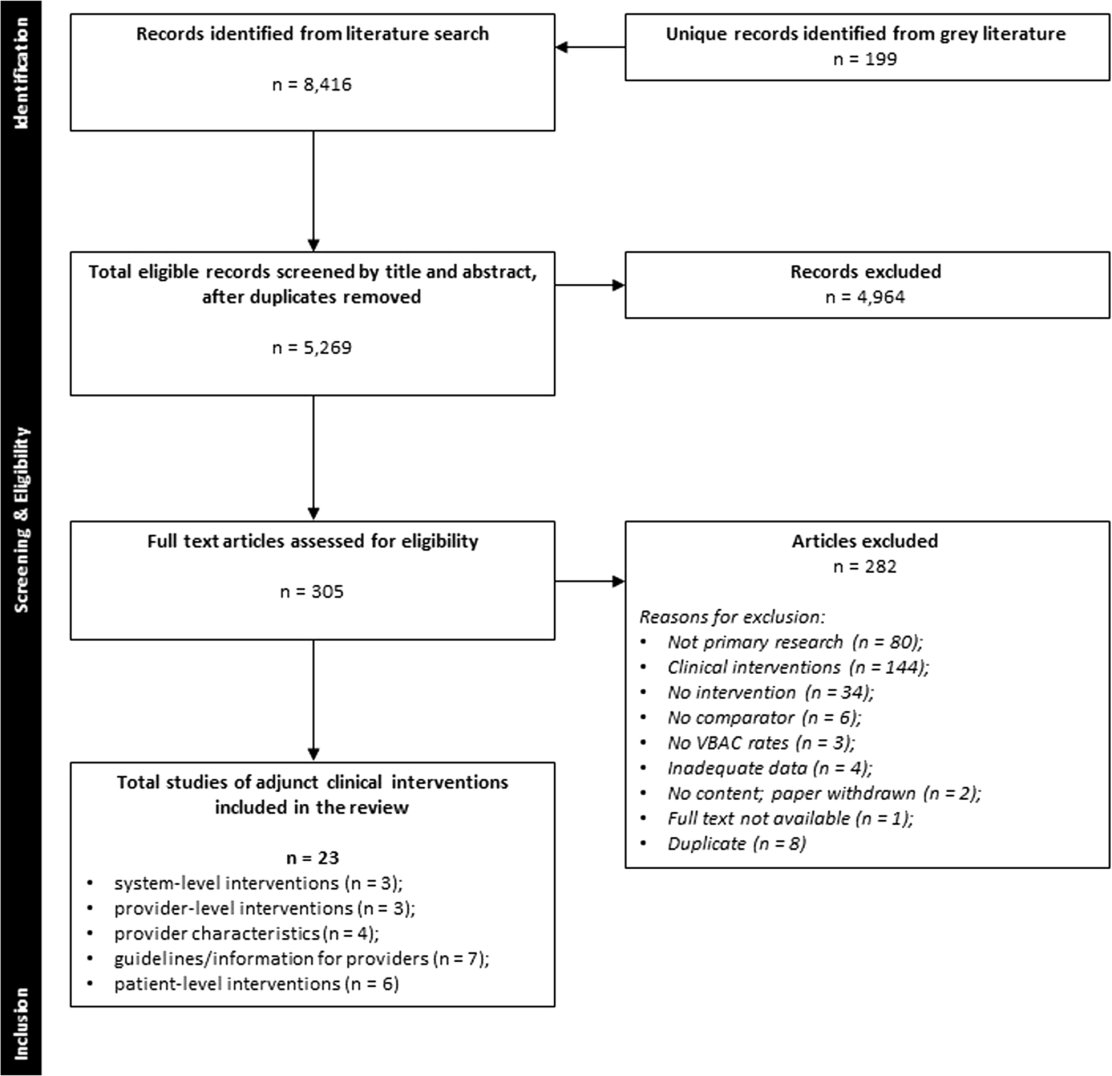
PRISMA flow of study selection

The literature search identified 5269 unique records eligible for inclusion. After screening titles and abstracts, 305 potentially relevant articles were identified. Full text screening yielded 23 studies [22–44] included in the review. The screening process is illustrated in Fig. 1.

Studies published from 1987 to 2017 were conducted in the United States (13 studies; 57% [23–27, 30, 34, 35, 37–39, 42, 44]), Canada (three studies; 13% [28, 32, 36]), United Kingdom (three studies; 13% [33, 40, 41]), Australia (one study; 4% [29]), China (one study [43]), Portugal (one study; 4% [22]), and Taiwan (one study; 4% [31]). Funding was from non-industry sources (ten studies; 43% [25–28, 30, 32, 33, 36, 40, 42]), without funding (two studies; 9% [22, 43]), or was not reported (11 studies; 48% [23, 24, 29, 31, 34, 35, 37–39, 41, 44]). The sample size varied depending on unit of reporting, with a mean of 1276 women (14 studies; [23, 25–30, 32, 33, 36, 40–43] range 96 to 4732) or 306,097 deliveries (nine studies; [22, 24, 31, 34, 35, 37–39, 44] range 5308 to 1,260,186).

The majority of studies included women (or records of women) who delivered in hospital (20 studies; [23–25, 27–34, 36–44] 87%), health clinics (one study; [26] 4%), and in multiple settings including hospitals and at home (two studies; [22, 35] 9%). Of the 12 studies [23, 25, 26, 28, 29, 32, 36, 39, 40, 42–44] (52%) that reported maternal age, a wide range of women (18 years and younger to 40 years and older) were represented. Studies explicitly reported including women with 1 prior cesarean delivery (six studies [26, 28, 29, 40–42]), one or two prior cesarean deliveries (one study [37]), and at least one prior cesarean delivery (three studies [23, 33, 36]); the latter three included some women with three or more prior cesarean deliveries.

While all studies reported the proportion of women who had a VBAC, about half (13 studies; [25, 27–30, 32, 34, 36, 37, 40–42, 44] 57%) provided comparative proportions of women who had a TOLAC.

Most studies were cohorts (16 studies; one non-concurrent cohort contained 3-arms [22, 23, 25, 29–31, 34–42, 44]); a small proportion were RCTs (five studies; three trials contained three-arms [26, 28, 32, 33, 43]), before-after (one study [24]), and cross-sectional (one study [27]).

Table 1 summarizes the strategies/outcome themes among the studies. Tables 2, 3, 4, 5 and 6 summarize the included studies by categories of interventions. Additional file 1: Appendix 2 details characteristics of the individual studies.

**Table 1:** Summary of adjunct clinical interventions of included studies

Intervention Category	Strategy/Outcome Themes	TOLAC^a^	VBAC^a^	Study (study design)
System-level	Education and training of providers	NA	+	Ayres-de-Campos 2015 (NCC)
	Targeted CD rates with hospital funding	NA	+	Ayres-de-Campos 2015 (NCC)
	Targeted VBAC rates with hospital funding	NA	+	Liu 2013 (NCC)
	Hospital peer-review of CD/VBAC	NA	NS;	Bickell 1996 (BA);
			+, NC	Liu 2013 (NCC)
Provider-level	Opinion leader VBAC	+	+	Lomas 1991 (RCT)
	Hospital with laborists	+	+NS	Feldman 2015 (CS)
	Second opinion requirement for all CDs	+	+	Myers 1993 (NCC)
Provider characteristics	Midwifery vs. non-midwifery provider	+	+	Zhang 2016 (RCT);
				White 2016 (NCC)
	Family physician vs. obstetrician	+	+	Russillo 2008 (CS)
	Night float call vs. traditional call	+	+	Yee 2017 (RC)
Provider guidelines/information	Education and management direction	+	+	Bellows 2016 (NCC);
				Kosecoff 1987 (RC);
				Sanchez-Ramos 1990 (NCC);
				Santerre 1996 (NCC);
		–	–	Pinette 2004 (NCC);
				Zweifler 2006 (NCC);
		NA	NC	Studnicki 1997 (NCC)
Patient-level	Obstetric information vs. no information	+	+	Wong 2014 (PC)
	Verbal vs. written patient information	+NS	+NS	Fraser 1997 (RCT);
	Dedicated VBAC clinic vs. standard care	NA	+	Gardner 2014 (NCC)
	Decision analysis (computerized) vs. brochures	NA	+NS	Eden 2014 (RCT)
	Decision analysis vs. information vs. usual care	NA	+NS	Montgomery 2007 (RCT)
	One-on-one antenatal VBAC counseling vs. standard care	+	–	Cleary-Goldman 2005 (PC)

*TOLAC* trial of labor after cesarean, *VBAC* vaginal birth after cesarean, *NCC* non-concurrent cohort, *CD(s)* cesarean delivery, *BA* before-after, *RCT* randomized clinical trial, *CS* cross-sectional, vs. versus, *RC* retrospective cohort, *PC* prospective cohort

^a^Rates reported as increased (+), increased but not statistically significant (+NS), decreased (−), no change (NC), or not applicable/not assessed (NA)

**Table 2:** Summary of studies – system-level interventions

Study; Design; Country, setting; Funding	Population; Study period	Intervention & comparator (no. of participants)			TOLAC rate^a^	VBAC rate^a^	VBAC/TOLAC rate^a^
Ayres-De-Campos (2015) Non-concurrent cohort Portugal, state-owned hospitals, private hospitals & home births No funding	All deliveries from state-owned hospitals, private hospitals & home births during study period January 1, 2000–September 30, 2014	Grp 1 (2000–2009): no concerted action (*n* = 913,219)	Grp 2 (2010–2014): concerted action by independent committee (visits to state—owned hospitals with CS rates > 35%; meetings with obstetric & midwifery staff; training courses) (*n* = 346,157)		NR	2000: 14,993 (14.5%); 2001: 13,298 (13.7%); 2002: 15,360 (15.8%); 2003: 13,890 (14.8%); 2004: 13,710 (15.0%); 2005: 13,147 (14.6%); 2006: 15,700 (17.9%); 2007: 15,431 (18.1%); 2008: 13,837 (16.2%); 2009: 13,399 (16.4%) vs. 2010: 14,834 (17.9%); 2011: 17,624 (22.8%); 2012: 18,076 (25.1%); 2013: 16,365 (25.8%); Jan–Sept 2014: 16,859 (32.8%)	NR
Bickell (1996) Controlled before-after US, hospitals with high/average/low cesarean rate Funding NR	Hospitals from eight designated Health Service Areas of New York State 1988 & 1993	I: reviewed hospitals, external peer reviews by ACOG trained team (audit & feedback) (45 hospitals; mean 1400–1500 deliveries)	C: non-reviewed hospitals, had an obstetric service (120 hospitals; mean 1700 deliveries)		NR	1988: I: mean 10.1 ± 1.4% C: mean 12.1 ± 0.9% NS (*p* > 0.01) 1993: I: mean 24.8 ± 2.0% C: mean 24.8 ± 1.1% NS (*p* > 0.01)	NR
Liu (2013) Non-concurrent cohort Taiwan, tertiary hospital Funding NR	All pregnant women delivering by cesarean section June 2001–August 2010	Period 1 (June 2001–July 2002): before implementation of budget systems (*n* = 800)	Period 2 (July 2002–August 2005): global budget system (*n* = 1887)	Period 3 (August 2005–2010): hospital-based self-management program (*n* = 2621)	NR	P1: 38 (4.8%) P2: 231 (12.2%) P3: 298 (11.4%) Period 1 vs. 2, *p* < 0.001 Period 2 vs. 3, *p* = 0.3950	NR

*no.* number, *TOLAC* trial of labor after cesarean, *VBAC* vaginal birth after cesarean, *Grp* group, *n* number, *CS* cesarean section, *NR* not reported, vs. versus, *US* United States, *I* intervention, *ACOG* American College of Obstetricians and Gynecologists, *C* comparator, *NS* not significant

^a^Results of statistical tests or summary statistics were extracted whenever these were reported within studies

**Table 3:** Summary of studies – provider-level interventions

Study; Design; Country, setting; Funding	Population; Study period	Intervention & comparator (no. of participants)		TOLAC rate^a^	VBAC rate^a^	VBAC/TOLAC rate^a^	Study; Design; Country, setting; Funding
Feldman (2015) Cross-sectional US, community hospitals Non-industry funded	Women with live-born, singleton, vertex gestations, with prior CS January 2012–January 2014	I: hospitals employing laborists (≥1 physician in hospital, primary focus is to care for patients in labor and delivery) (*n* = 2621)		C: hospitals without laborists (*n* = 2111)	I: 356 (13.6%) C: 201 (9.5%), *p* = 0.0318	I: 253 (9.7%) C: 137 (6.5%), *p* = 0.0302	I: 253 (71.0%) C: 136 (67.9%), *p* = 0.2943
Lomas (1991) RCT, 3-arm Canada, community hospitals Non-industry funded	Study site inclusion needed 100 beds (10+ obstetrical), no status as teaching institution, not in county with teaching institution 1988–1989	I 1: audit and feedback (*n* = 524)	I 2: opinion leader education (*n* = 739)	C: eight control hospitals, practice guideline mailed to obstetrical care (*n* = 1233)	I 1: 112 (21.4%) I 2: 282 (38.2%) C: 349 (28.3%), *p* = 0.007	I 1: 62 (11.8%) I 2: 187 (25.3%) C: 179 (14.5%), *p* = 0.003	I 1: 62 (55.4%) I 2: 187 (66.3%) C: 179 (51.3%)
Myers (1993) Follow-up to non-concurrent cohort (1985–1987) US, level 3 prenatal center Funding NR	All patients in obstetric department 1985–1991	Grp 1 (1985): before hospital initiative (*n* = 122)		Grp 2 (1986–1991): after hospital initiative; 2nd opinion required for all CS, VD was preferred, dystocia accepted as indication for CD (*n* = 1840)	1985: 55 (45.0%) vs. 1986: 132 (68.4%) 1987: 233 (86.0%) 1988: 243 (88.3%) 1989: 255 (91.3%) 1990: 312 (85.4%) 1991:374 (81.8%)	1985: 29 (23.8%) vs. 1986: 106 (54.9%) 1987: 162 (59.8%) 1988: 167 (60.1%) 1989: 188 (67.4%) 1990: 242 (66.3%) 1991: 291 (63.7%)	1985: 29 (52.7%) vs. 1986: 106 (80.3%) 1987: 162 (69.5%) 1988: 167 (73.7%) 1989: 188 (73.7%) 1990: 242 (77.5%) 1991: 291 (77.8%)

*no.* number, *TOLAC* trial of labor after cesarean, *VBAC* vaginal birth after cesarean, *US* United States, *CS* cesarean section, *I* intervention, *C* comparator, *RCT* randomized controlled trial, *NR* not reported, *Grp* group, *VD* vaginal delivery, *CD* cesarean delivery

^a^Results of statistical tests or summary statistics were extracted whenever these were reported within studies

**Table 4:** Summary of studies – provider characteristics

Study; Design; Country, setting; Funding	Population; Study period	Intervention & comparator (no. of participants)		TOLAC rate^a^	VBAC rate^a^	VBAC/TOLAC rate^a^
Russillo (2008) Cross-sectional Canada, secondary care urban hospital Non-industry funded	Pregnant women with at least one previous CS, singleton delivery, birth weight at least 500 g January 1995–December 2003	Grp 1: deliveries performed by obstetricians (*n* = 3493)	Grp 2: deliveries performed by family physicians (*n* = 201)	Grp 1: 1768 (50.6%) Grp 2: 163 (81.1%), *p* < 0.001	Grp 1: 1136 (32.5%) Grp 2: 124 (61.7%)	Grp 1: 1136 (64.3%) Grp 2: 124 (76.1%), *p* = 0.002
White 2016 Non-concurrent cohort UK, tertiary teaching hospital Non-industry funded	Women with one previous CS who received antenatal and intrapartum care during study period 2008 & 2011	Grp 1 (2008): obstetrician-led antenatal care (*n* = 209)	Grp 2 (≥2011): midwife-led antenatal care (*n* = 196)	Attempted VBAC: Grp 1: 143 (68.4%) Grp 2: 153 (78.1%)	Actual VBAC: Grp 1: 98 (46.9%) Grp 2: 120 (61.2%); aOR 1.79 (95% CI 1.17–2.75), *p* < 0.05 Spontaneous VBAC: Grp 1: 67 (32.1%) Grp 2: 85 (43.4%); OR 1.62 (95% CI 1.08–2.43)	Successful/attempted VBAC: Grp 1: 98 (68.5%) Grp 2: 120 (78.4%); OR 1.67 (95% CI 0.99–2.82), NS (*p* > 0.05)
Yee 2017 Retrospective cohort US, large teaching hospital Funding NR	Women ≥18 years old with one prior low transverse CD, a term, cephalic singleton gestation, and no prior VD January 2008–June 2013	Grp 1: night float schedule (*n* = 556)	Grp 2: traditional call schedule (*n* = 946)	Grp 1: 184 (33.1%) Grp 2: 156 (16.5%), *p* < 0.001	Grp 1: 104 (18.7%) Grp 2: 88 (9.3%), *p* < 0.001	Grp 1: 104 (56.5%) Grp 2: 88 (56.4%), *p* = 0.98
Zhang 2016 RCT China, hospital obstetric department No funding	Women with a history of previous CS in labor willing to undergo a VD May 2013–November 2014	I: continuing midwifery care (*n* = 48)	C: standard maternity care (*n* = 48)	NR	I: 42 (87.5%) C: 32 (66.7%), *p* < 0.05	NR

*no.* number, *TOLAC* trial of labor after cesarean, *VBAC* vaginal birth after cesarean, *CS* cesarean section, *g* grams, *Grp* group, *UK* United Kingdom, *aOR* adjusted odds ratio, *CI* confidence interval, *OR* odds ratio, *NS* not significant, *US* United States, *CD* cesarean delivery, *VD* vaginal delivery, *RCT* randomized controlled trial, *I* intervention, *C* comparator, *NR* not reported

^a^Results of statistical tests or summary statistics were extracted whenever these were reported within studies

**Table 5:** Summary of studies – guidelines or information for providers

Study; Design; Country, setting; Funding	Population; Study period	Intervention & comparator (no. of participants)		TOLAC rate^a^	VBAC rate^a^	VBAC/TOLAC rate^a^
Bellows (2016) Non-concurrent cohort US, tertiary care academic hospital Funding NR	All women who underwent TOLAC, at least one prior CD & live, singleton gestation in cephalic presentation, 24 0/7 weeks of gestation July 1, 2009-December 31, 2013	Grp 1 (2009–2011): pre-2011 guideline (*n* = 450)	Grp 2 (2011–2013): post-2011 guideline implementation (offering TOLAC; inducing labor; administering oxytocin) (*n* = 781)	NR	Grp 1: NR (26.0%) Grp 2: NR (33.3%)	Grp 1: 351 (78.1%) Grp 2: 616 (78.9%), *p* = 0.75
Kosecoff (1987) Retrospective cohort US, acute, non-specialty, nonfederal hospitals > 150 beds. Non-industry funded	Women with previous low transverse CS January 1979–December 1979 (time 1) January 1980–September 1980 (time 2) July 1981–June 1982 (time 3)	Period 1 (January–December 1979) & Period 2 (January–September 1980): before NIH Consensus Development conference recommendations (*n* = 35 & *n* = 64)	Period 3 (1981–1982): after conference recommendations; women should be given TOLAC for potential VD (*n* = 70)	Period 1: 2 (5.7%) Period 2: 7 (10.9%) vs. Period 3: 20 (28.6%)	Period 1: 2 (5.7%) Period 2: 4 (6.3%) vs. Period 3: 11 (15.7%)	Period 1: 2 (100%) Period 2: 4 (57.1%) vs. Period 3: 11 (55.0%)
Pinette (2004) Non-concurrent cohort US, birth certificate & hospital reported data Funding NR	All women with previous CS giving birth at 20 weeks of gestation or more 1998–2001	Grp 1 (1998): pre-exposure (birth certificate *n* = 1410; hospital data *n* = 1386)	Grp 2 (1999–2001): ACOG guideline revision (birth certificate data *n* = 4463; hospital data *n* = 4015)	NR	Birth certificate data: 1998: 424 (30.1%) vs. 1999: 327 (22.6%) 2000: 277 (17.9%) 2001: 193 (13.1%) 1988 vs. 2001, RR 2.8 (95% CI 2.5–3.2), *p* < 0.01 Hospital-reported data: 1998: 489 (35.3%) vs. 1999: 411 (28.2%) 2000: 321 (23.1%) 2001: 156 (13.3%) RR 3.5 (95% CI 3.1–4.2), *p* < 0.01	NR
Sanchez –Ramos (1990) Non-concurrent cohort US, regional perinatal center Funding NR	Women with one or two previous CS, with low transverse or vertical scars not extending into uterine corpus 1986–1989	Grp 1 (1986–1987): before July 1, 1987 department-wide guideline change (*n* = 899)	Grp 2 (1988–1989): after July 1, 1987, new guidelines for intrapartum management (*n* = 1105)	1986: 139 (31.7%) 1987: 193 (41.9%) vs. 1988: 402 (76.6%) 1989: 487 (84.0%); Difference: 52.2%, *p* < 0.0001	1986: 90 (20.5%) 1987: 142 (30.8%) vs. 1988: 342 (65.1%) 1989: 403 (69.5%); Difference: 48.9%; *p* < 0.0001	1986: 90 (64.7%) 1987: 142 (73.6%) vs. 1988: 342 (85.1%) 1989: 403 (82.8%); Difference: 18.0%; *p* < 0.0001
Santerre (1996) Non-concurrent cohort US, hospitals Funding NR	Women with a previous CS 1987–1991	Grp 1 (before 1987–1988): before practice guideline implementation (*n* = NR)	Grp 2 (after Oct. 1988): ACOG practice guideline, prior cesarean section no longer a reason for repeat cesarean (*n* = NR)	NR	VBAC rate in US (data for Massachusetts hospitals NR) 1985: 6.6% 1986: 8.5% 1987: 9.8% 1988: 12.6% vs. 1989: 18.5% 1990: 20.4% 1991: 24.2% 1992: 25.1% 1993: 25.4%	NR
Studnicki (1997) Non-concurrent cohort US, nonfederal acute care provider hospitals Funding NR	Women with prior CS 1990–1993	Grp 1 (1990–1992): before practice guidelines (*n* = 66,702)	Grp 2 (1993): after legislatively imposed practice guidelines (*n* = 23,142)	NR	1990: 4816 (21.8%) 1991: 5540 (25.6%) 1992: 6133 (26.7%) vs. 1993: 7151 (30.9%)	NR
Zweifler (2006) Non-concurrent cohort US, California Department of Health Services Birth Statistical Master Files Funding NR	Women who previously gave birth by cesarean delivery & had singleton birth planned in a California hospital 1996–2002	Grp 1 (1996–1999): before ACOG VBAC guideline revision (*n* = NR^b^)	Grp 2 (2000–2002): after ACOG VBAC guideline revision (*n* = NR^b^)	Attempted VBAC: Grp 1: NR (24.0%) Grp 2: NR (13.5%) Difference: 44% decrease, *p* < 0.001	1996–2002: 61,684 (16.0%)	NR

*no.* number, *TOLAC* trial of labor after cesarean, *VBAC* vaginal birth after cesarean, *US* United States, *NR* not reported, *CD* cesarean delivery, *Grp* group, *CS* cesarean section, *NIH* National Institute of Health, *VD* vaginal delivery, vs. versus, *ACOG* American College of Obstetricians and Gynecologists, *RR* relative risk, *CI* confidence interval

^a^Results of statistical tests or summary statistics were extracted whenever these were reported within studies

^b^Study reported number of live births separately from number of women; table reflects data for number of women whenever this was reported

**Table 6:** Summary of studies – patient-level interventions

Study; Design; Country, setting; Funding	Population; Study period	Intervention & comparator (no. of participants)		TOLAC rate^a^	VBAC rate^a^	VBAC/TOLAC rate^a^	Population; Study period
Cleary-Goldman (2005) Prospective cohort with controls US, tertiary care center Non-industry funded	Women eligible for a TOLAC 12-month period	I: one-on-one VBAC counselling, in 2nd and 3rd trimesters (*n* = 95)		C: no extra counselling, standard care (*n* = 221)	I: 44 (46.3%) C: 85 (38.5%)	I: 26 (27.4%) C: 70 (31.7%)	I: 26 (59.1%) C: 70 (82.4%)
Eden (2014) RCT US, clinics Non-industry funded	Pregnant women with one prior CS, 18 years or older, pregnant with one fetus, low transverse uterine scar, and providers had given option of TOLAC September 17, 2005-May 4, 2007	I: Evidence-based, interactive decision aid (*n* = 66)		C: two evidence-based educational brochures about cesarean delivery and VBAC (*n* = 65)	NR	I: NR (41.0%) C: NR (37.0%), *p* = 0.724	NR
Fraser (1997) RCT Canada, hospitals Non-industry funded	Women with single previous low transverse CS, gestational age < 28 weeks. April 1992–November 1994	I: Verbal prenatal education program – pamphlet + 2 individualized contacts (*n* = 641)		C: Document prenatal education program – written information (*n* = 634)	I: 465 (72.5%) C: 440 (69.4%); RR 1.1 (95% CI 1.0–1.1)	I: 339 (52.9%) C: 310 (48.9%); RR 1.1 (95% CI 1.0–1.2)	I: 339 (72.9%) C: 310 (70.5%)
Gardner (2014) Non-concurrent cohort Australia, metropolitan teaching hospital Funding NR	Women with a single prior CS, presenting in their next pregnancy 2006 (before) & May 2009–October 2010	Grp 1 (2006): routine care, counselling for mode of birth on ad-hoc basis (*n* = NR)		Grp 2 (2009–2010): two combined management strategies – Risk Associated Pregnancy consultant & NBAC clinic (*n* = 396)	Grp 1: NR Grp 2: 164 (41.4%)	VBAC rate for NBAC Grp 1: NR (17.2%) Grp 2: 107 (27.0%), *p* < 0.001	Grp 1: NR Grp 2: 107 (65.2%)
Montgomery (2007) RCT UK, maternity units Non-industry funded	Pregnant women with one previous lower segment CS, delivery expected at ≥37 weeks; most recent delivery is cesarean. May 2004–August 2006	I 1: decision analysis aid (*n* = 235)	I 2: information program (*n* = 241)	C: usual care (*n* = 239)	NR	I 1: 88 (37.4%) I 2: 70 (29.2%) C: 72 (30.3%) I 1 vs. C: aOR 1.42 (95% CI 0.94–2.14), *p* = 0.22 I 2 vs. C: aOR 0.93 (95% CI 0.61–1.41), *p* > 0.9 I1 vs. I2: aOR 1.53 (95% CI 1.01–2.30), *p* = 0.11	NR
Wong (2014) Prospective cohort UK, district general hospital Funding NR	Women with one previous lower segment CS, no contraindications for VBAC 12-month period commencing January 1, 2012	I: one-stop obstetrician-led cesarean education and antenatal sessions (OCEANS) (*n* = 188)		C: did not attend OCEANS (*n* = 78)	I: 108 (57.4%) C: 33 (42.3%), *p* = 0.02	I: 59 (31.4%) C: 20 (25.6%)	I: 59 (54.6%) C: 20 (60.6%) *p* = 0.69†

*no.* number, *TOLAC* trial of labor after cesarean, *VBAC* vaginal birth after cesarean, *US* United States, *I* intervention, *C* comparator, *RCT* randomized controlled trial, *CS* cesarean section, *NR* not reported, *RR* relative risk, *CI* confidence interval, *Grp* group, *NBAC* next birth after cesarean, *UK* United Kingdom, *aOR* adjusted odds ratio, *OCEANS* obstetrician-led cesarean education and antenatal session

^a^Results of statistical tests or summary statistics were extracted whenever these were reported within studies

†study reports difference as *p* = 0.69 (Table 2) and *p* = 0.55 (abstract)

### Methodological quality of included studies

All of the studies received a score for having a clear research question or objective, and for collecting data that addressed the intended research question.

Of the five RCTs, four (80%) [26, 28, 32, 33] described the randomization process clearly, but only one (20%) [33] clearly described allocation concealment or blinding. Four (80%) RCTs [28, 32, 33, 43] had complete outcome data for at least 80% of the participants. Three (60%) RCTs [26, 28, 33] had a withdrawal or drop-out rate of less than 20%. Overall, one (25%) RCT [33] met all of the criteria (four out of four stars).

The majority (16 studies; 89%) [22–24, 27, 29–31, 35–42, 44] of the non-randomized controlled studies recruited participants or organizations in a way that minimized selection bias. Most (17 studies; 94%) [22–25, 27, 30, 31, 34–42, 44] studies used appropriate measurements for the interventions/exposures and outcomes, and used interventions that did not present potential contamination between groups. Only seven (39%) studies [23, 24, 27, 39, 40, 42, 44] accounted for important differences between groups, or controlled for such differences in the data analysis. Many studies (*n* = 16; 89%) [22–25, 27, 29, 31, 34–40, 42, 44] had complete outcome data for at least 80% of the participants, or an acceptable response or follow-up rate (i.e., 60% or above). Overall, seven (39%) [23, 24, 27, 39, 40, 42, 44] studies scored four stars (out of four).

Methodological quality assessments are summarized in Table 7; detailed study assessments are in Additional file 1: Appendix 3.

**Table 7:** Summary of methodological quality of included studies

MMAT^a^ criteria	Screening questions		Quantitative/ control group				Quantitative non-randomized				Total^b^
Study	Clear research questions or objectives?	Do collected data address the research questions/objective?	2.1 Clear description of randomization?	2.2 Clear description of allocation concealment (or blinding)?	2.3 Complete outcome data (≥80%)?	2.4 Low withdrawal/drop-out (< 20%)?	3.1 Participants/organizations recruitment - minimizes selection bias?	3.2 Appropriate measurements used for intervention & outcomes?	3.3 Participants/organizations comparable, or are differences accounted for?	3.4 Complete outcome data (80% or above) or acceptable follow-up rate?	
Ayres-De-Campos (2015)	✰	✰	NA	NA	NA	NA	✰	✰	–	✰	✰✰✰ (75%)
Bickell (1996)	✰	✰	NA	NA	NA	NA	✰	✰	✰	✰	✰✰✰✰(100%)
Bellows (2016)	✰	✰	NA	NA	NA	NA	✰	✰	✰	✰	✰✰✰✰(100%)
Cleary-Goldman (2005)	✰	✰	NA	NA	NA	NA	–	✰	–	✰	✰✰ (50%)
Eden (2014)	✰	✰	✰	–	–	✰	NA	NA	NA	NA	✰✰ (50%)
Feldman (2015)	✰	✰	NA	NA	NA	NA	✰	✰	✰	✰	✰✰✰✰(100%)
Fraser (RCT)	✰	✰	✰		✰	✰	NA	NA	NA	NA	✰✰✰ (75%)
Gardner (2014)	✰	✰	NA	NA	NA	NA	✰	–	–	✰	✰✰ (50%)
Kosecoff (1987)	✰	✰	NA	NA	NA	NA	✰	✰	–	–	✰✰ (50%)
Liu (2013)	✰	✰	NA	NA	NA	NA	✰	✰	–	✰	✰✰✰ (75%)
Lomas (1991)	✰	✰	✰	–	✰	–	NA	NA	NA	NA	✰✰ (50%)
Montgomery (2007)	✰	✰	✰	✰	✰	✰	NA	NA	NA	NA	✰✰✰✰(100%)
Myers (1993)	✰	✰	NA	NA	NA	NA	–	✰	–	✰	✰✰ (50%)
Pinette (2004)	✰	✰	NA	NA	NA	NA	✰	✰	–	✰	✰✰✰ (75%)
Russillo (2008)	✰	✰	NA	NA	NA	NA	✰	✰	–	✰	✰✰✰ (75%)
Sanchez-Ramos (1990)	✰	✰	NA	NA	NA	NA	✰	✰	–	✰	✰✰✰ (75%)
Santerre (1996)	✰	✰	NA	NA	NA	NA	✰	✰	–	✰	✰✰✰ (75%)
Studnicki (1997)	✰	✰	NA	NA	NA	NA	✰	✰	✰	✰	✰✰✰✰(100%)
White (2016)	✰	✰	NA	NA	NA	NA	✰	✰	✰	✰	✰✰✰✰(100%)
Wong (2014)	✰	✰	NA	NA	NA	NA	✰	✰	–	–	✰✰ (50%)
Yee (2017)	✰	✰	NA	NA	NA	NA	✰	✰	✰	✰	✰✰✰✰(100%)
Zhang (2016)	✰	✰	–	–	✰	–	NA	NA	NA	NA	✰ (25%)
Zweifler (2016)	✰	✰	NA	NA	NA	NA	✰	✰	✰	✰	✰✰✰✰(100%)

^a^ Assessed using the Mixed Methods Appraisal Tool; ^b^ Total score is out of four stars (✰✰✰✰), whereby each assessment criterion met by a study was awarded a star (✰), and a criterion not met by a study was marked with a dash (−); *NA* not applicable

### TOLAC and VBAC rates

#### System-level interventions

Three studies [22, 24, 31] examined system-level interventions (Table 2). One non-concurrent cohort compared deliveries in continental Portugal before and after a concerted action to reduce cesarean section rates based on transmission and training of healthcare professionals as well as targeted cesarean delivery rates for contingency-based hospital funding, and found an increase in the VBAC rate from 16.4% (13,399 of 81,750 VBACs) in 2009 to 32.8% (16,859 of 51,478 VBACs) in 2014 (*p* < 0.001) [22]. Another non-concurrent cohort of deliveries at a tertiary hospital in Taiwan found that rates of vaginal deliveries in women with previous cesarean deliveries increased (from 4.8% [38 of 800 deliveries] to 12.2% [231 of 1887 deliveries]) after implementation of direct government funding of hospitals from 2002 to 2005 (rate ratio 0.82, 95% CI 0.74–0.90, *p* = 0.0001), but the rate did not improve further (from 12.2% [231 of 1887 deliveries] to 11.4% [298 of 2621 deliveries]) with the additional employment of a hospital-based post-operative peer review and audit strategy from 2005 to 2010 (rate ratio 0.98, 95% CI 0.96–0.99, *p* = 0.0003) [31]. One study compared peer reviewed with non-reviewed hospitals (45 [mean 1430 deliveries in 1988 and mean 1503 deliveries in 1993] versus 120 hospitals [mean 1720 deliveries in 1988 and 1993]), and found that VBAC rates increased between the years by 14.6 and 12.7% (reviewed and non-reviewed hospitals, respectively), although the difference between reviewed and non-reviewed hospitals was not statistically significant [24].

#### Provider-level interventions

Three studies examined provider-level interventions [27, 32, 34] (Table 3). One RCT of community hospitals compared opinion leader education (739 women from four hospitals) and audit and feedback (524 women from four hospitals) to mailed guideline recommendations (1233 women from eight hospitals), and found that women were more likely to attempt a TOLAC when delivering in obstetric departments with influential opinion leaders (38.2%) compared to units with audit and feedback (21.4%) or mailed practice guidelines (28.3%) (46% higher in the opinion leaders group versus the other groups, *p* = 0.007) [32]. Women were also more likely to have VBACs in the opinion leader group (25.3%) compared with the audit and feedback (11.8%) and guideline groups (14.5%) (85% higher in the opinion leaders group versus the other groups, *p* = 0.003) [32]. A cross-sectional study found that a higher proportion of women with prior cesarean delivery had a TOLAC in hospitals employing laborists (356 of 2621 women; 13.6%) compared with hospitals without laborists (201 of 2111 women; 9.5%) [27]. A higher rate of successful VBACs occurred in the same group, however the result was not statistically significant (9.7% versus 6.5%; adjusted odds ratio (aOR) 1.10, 95% CI 0.82–1.47, *p* = 0.5417) [27]. A non-concurrent cohort comparing deliveries in 1985 with deliveries in 1986 to 1991 after the implementation of a hospital initiative utilizing a second opinion by an obstetrician for primary and repeat cesarean deliveries, found increased rates of TOLAC (45.0% versus range 68.4 to 91.3%, pre- versus post-intervention, respectively) and VBAC (23.8% versus range 54.9 to 67.4%, pre- versus post-intervention, respectively) [34].

#### Provider characteristics

Four studies [36, 40, 42, 43] examined the effect of provider characteristics on VBAC rates (Table 4). A small RCT of women in labor compared midwifery care (*n* = 48) with standard maternity care (*n* = 48); the authors reported a higher proportion of VBAC among women receiving continuous midwifery care from the antenatal to postnatal period (87.5% versus 66.7% of women, *p* < 0.05) [43]. Another study examined midwifery care and found that among women with one previous cesarean delivery, there was a higher rate of attempted VBACs in the post-intervention (midwifery-led antenatal care; 153 of 196 women; 78.1%) compared with the pre-intervention group (traditional obstetrician-led antenatal care; 143 of 209 women; 68.4%) [40]. More VBACs occurred in the group who received care from a midwife in 2011 (120 of 196 women; 61.2%) than among women who received obstetrician-led antenatal care in 2008 (98 of 209 women; 46.9%, aOR 1.79; 95% CI 1.17–2.75, *p* < 0.05). One retrospective cohort compared physicians with a traditional call schedule (946 women) with physicians on a night float call schedule (556 women); eligible women were more likely to undergo a TOLAC when delivered by physicians on a night float call system (OR 2.50, 95% CI 1.96–3.20, *p* < 0.001) and the effect persisted when the groups were adjusted for body-mass index (BMI), gestational age (GA) and physician (aOR 2.64, 95% CI 1.65–4.25, *p* < 0.001) [42]. A cross-sectional study of women with at least one previous cesarean delivery with a singleton delivery compared women delivered by an obstetrician (*n* = 3493) with women delivered by a family physician (*n* = 201), and found that more TOLACs occurred in the latter than the former group (81.1% versus 50.6%, *p* < 0.001) as well as VBACs (61.7% versus 32.5%) [36].

#### Guidelines or information for providers

Seven non-concurrent cohort studies in the US [23, 30, 35, 37–39, 44] examined VBAC rates before and after guidelines or information for providers were implemented (Table 5). Kosecoff et al. compared VBAC rates in 1979 and 1980 (35 and 64 women, respectively) before the National Institutes of Health conference recommendations, with rates in 1981 to 1982 (70 women); a greater proportion of women had a TOLAC (5.7 and 11.0% pre- versus 28.6% post-recommendations) and a VBAC (5.7 and 6.3% pre- versus 15.7% post-recommendations) after the conference recommendations (adjusted positive linear trend of 2.4 [5.8%] for TOLAC and 2.1 [4.5%] for VBAC) [30]. Another study comparing before (1987–1988) and after (1988–1991) the ACOG practice guidelines were implemented reported that VBAC rates increased by 5.6 percentage points as a result of the guideline and its information dissemination [38]. Pinette et al. also compared rates of VBAC before (1998) and after (1999–2001) ACOG guidelines were revised to require the presence of surgical personnel throughout a trial of labour, and found a marked decline in hospital VBAC data (relative risk 3.5, 95% CI 3.1–4.2, *p* < 0.01), citing factors such as patient refusal post-counselling, inability of institutions to meet requirements, and lack of support from the obstetric service [35]. Zweifler et al. examined the effect of the ACOG revision to provide immediate cesarean capability (1996 to 1999 versus 2000 to 2002, before versus after, respectively) and found that there were comparatively fewer TOLACs (24.0% before versus 13.5% after guideline revision, *p* < 0.001) and successful VBACs among women with TOLACs (82.8% [41,961 of 50,670 deliveries] before versus 81.8% [19,273 of 23,573 deliveries] after guideline revision) [44]. In a study focused on the impact of state-legislated practice guidelines, the authors reported that dissemination alone did not significantly increase the VBAC rate (7151 of 23,142 deliveries; 30.9% post-guideline in 1993) compared with the years leading up to the change (4816 of 22,091 deliveries [21.8%] in 1990; 5540 of 21,461 deliveries [25.6%] in 1991; and, 6133 of 22,970 deliveries [26.7%] in 1992) [39]. A study compared intrapartum management of women with prior cesareans before (1986–1987) and after (1988–1989) hospital guideline changes incorporated centralized decision-making, and found that rates of TOLAC increased from 31.7% (139 out of 438 women in 1986) to 84.0% (487 out of 580 women in 1989; *p* < 0.0001), and that the proportion of these women with subsequent VBACs also increased (from 64.7% [90 out of 139 women in 1986] to 82.8% [403 out of 487 women in 1989, *p* < 0.0001) [37]. Bellows et al. examined changes to hospital policies for TOLAC eligibility and labor induction guidelines; the authors reported that while the “overall VBAC rate” (number of women with a prior cesarean who had a VBAC) increased (26.0% pre- versus 33.0% post-guidelines, *p* < 0.0001), the “VBAC rate” (number of women who underwent a TOLAC and had a successful VBAC) was unchanged (78.9% pre- versus 78.1% post-guidelines, *p* = 0.75) [23].

### Patient-level interventions

Six studies [25, 26, 28, 29, 33, 41] examined patient-level interventions (Table 6). One RCT of women with a single previous low transverse cesarean delivery compared a verbal prenatal education program (641 women) with a written prenatal education program (634 women) and found no evidence of a clinically significant difference for TOLAC rate (72.5% versus 69.4%; relative risk 1.1, 95% CI 1.0–1.1) or VBAC rate (52.9% versus 48.9%; relative risk 1.1, 95% CI 1.0–1.2) [28]. Another RCT compared two interventions (decision analysis aid [235 women] and information program [241 women]) with usual care (239 women), reporting the highest rate of VBAC in the decision analysis group (37.4% versus 29.2% versus 30.3%, decision analysis versus information program versus usual care, respectively; no significant differences between groups) and concluded that women who received any decision aid had greater knowledge and less anxiety than women receiving standard obstetric care [33]. Another trial compared an evidence-based computerized decision aid (66 women) with evidence-based educational ACOG brochures (65 women) and reported that women experienced less decisional conflict in the former group compared with the latter, however, there was no significant difference in VBACs (41.0% versus 37.0%, *p* = 0.724) [26]. A cohort study of patient satisfaction with mode of delivery found that women who received formal one-on-one antenatal counseling (*n* = 95) had comparatively higher rates of TOLAC (46.3% versus 38.5%) but lower rates of VBAC (27.4% versus 31.7%) than women who didn’t participate in VBAC counseling (*n* = 221) [25]. A non-concurrent cohort of women with a single prior cesarean delivery presenting in their next pregnancy had a higher VBAC rate after the implementation of standardized consultant labor management with a dedicated antenatal clinic (27.0% versus 17.2%, *p* < 0.001) compared with women who received routine antenatal care with mode of birth counseling on an ad-hoc basis [29]. Another cohort study reported higher rates of TOLAC (57.4% versus 42.0%, *p* = 0.02) and VBAC (31.4% versus 25.6%) among women who attended an obstetrician-led cesarean delivery education and antenatal session (*n* = 188) compared with women who chose not to attend the session (*n* = 78), although the authors concluded that the overall rate of successful vaginal deliveries among women who attempted VBAC was not influenced by the education session [41].

## Discussion

This systematic review of adjunct clinical interventions aimed at influencing the rate of VBACs identified 23 studies which suggest that some provider-level interventions (e.g. opinion leader education in hospitals, employing laborists as providers, and utilizing obstetrician ‘second opinion’ for all cesarean deliveries), and provider characteristics (i.e., midwifery-led antenatal care, physicians working a night call float schedule, and birth deliveries by a family physician) are associated with higher TOLAC and VBAC rates, while system-level interventions (i.e., education and training of healthcare providers, contingency-based funding for delivery rates, and peer review/audit), patient-level interventions (i.e., different modes of information delivery, and antenatal counseling for women) and provider guidelines/information report mixed findings. The significant study heterogeneity in research designs, interventions and outcomes did not allow for a meta-analysis to be completed.

Other systematic reviews of adjunct clinical interventions to increase VBAC rates have reported similar findings, although eligibility criteria among these reviews differed slightly from the present study. Catling-Paull et al. [45] examined non-clinical interventions (27 studies) and concluded that local guidelines, opinion leaders and individualized information for women can impact the uptake and/or success of VBAC. While the present study also found that opinion leaders increased VBACs, evidence from guidelines had conflicting findings and information for women did not show significant differences between groups. Lundgren et al. [46] evaluated clinician-centered interventions designed to increase VBAC rates (three studies) and concluded that educational strategies delivered by opinion leaders significantly increased VBAC rates, while external peer review and audit and feedback had no significant effect; the present review also found the impact of opinion leader education on VBACs. A systematic review of women-centered interventions to increase VBACs (three studies) concluded that while decision aids and information programs during pregnancy did not appear to affect the rate of VBAC, they reduced women’s decisional conflict and increased their knowledge regarding birth options [47]. The present study echoed the findings that information for women was associated with increased VBAC in one study but without significant difference in two studies.

### Strengths and limitations of study

A methodologically rigorous systematic review of the literature was undertaken to capture a broad range of studies of adjunct clinical interventions directed at increasing maternal VBAC rates. However, several factors limit our confidence in effects of interventions, such as inclusion of non-randomized study designs, small number of studies per intervention category, and inconsistent results across heterogeneous studies. Moreover, many studies did not report important maternal baseline characteristics in a consistent manner, including antenatal history (e.g., parity, number of previous cesarean deliveries and vaginal births, medical history/risk factors) or indications (e.g., age, gestational age, fetal risk factors).

### Implications for practice

Based on the available evidence, attempts to increase vaginal births among women with prior cesareans need to incorporate different types of provider or provider-level interventions to achieve a greater likelihood of success. Hospitals that utilize the ‘influential opinion leader model’ to educate colleagues and patients can effect a behavior/clinical change by offering more women the opportunity of a VBAC. Staffing community hospitals with laborists may encourage more support for women to attempt a vaginal delivery. Requiring an expert second opinion prior to a cesarean may decrease the proportion of women who undergo cesarean deliveries (exclusive of those performed for acute emergencies) through counseling, thereby increasing choice and the number of attempted VBACs. Low risk maternity providers (e.g., midwives, family physicians) or the continuity of midwifery care may provide women with support and confidence to undergo a VBAC through a personalized and responsive approach. Overall, these are aligned with the SOGC recommendation that women be given the opportunity to consult with her obstetric care provider on the risks and benefits of TOLAC as well as awareness of availability of hospital resources for an elective cesarean section if indicated [12]. Adoption of any strategy or intervention to increase rates of TOLAC and subsequent VBAC must carefully weigh the potential benefits against the possible risks for mother and baby.

As the scope of the current review was sufficiently broad, the authors did not search for studies that examined barriers to VBAC or factors related to women’s motivations, preferences or decision-making. Studies of clinicians’ and women’s perspectives may provide insight on system, provider-level, and patient-oriented factors that influence rates of attempted and successful VBAC [48, 49]. For example, a qualitative study examined barriers associated with the ACOG VBAC guidelines and found that fear of liability affected the willingness of midwives and obstetricians in offering VBAC [6]. Other factors such as the continual presence of a physician, travel distance to a hospital that offers TOLAC (although 56% of California hospitals permit TOLAC, significantly fewer VBACs were actually carried out), and hospital policy for patient TOLAC eligibility presented as other systemic barriers restricting women’s access to a TOLAC [50]. Additionally, there is a paucity of studies on supports intended to facilitate shared decision-making between women and their healthcare providers [51]. Such evidence may provide context for effectiveness, acceptability and feasibility of interventions aimed at individual patients’ needs, decisions and satisfaction regarding mode of birth.

## Conclusion

This ‘up-to-date’ systematic review evaluated adjunct clinical interventions directed at increasing the rate of vaginal delivery among women with a prior cesarean delivery and provides evidence that some provider-level interventions and provider characteristics are associated with higher maternal TOLAC and VBAC rates. Further research, using robust study designs with documentation of population characteristics, is needed to provide stronger outcome evidence for the use and effect of adjunct clinical interventions. Enhancing the woman’s education and her opportunity to consider and choose VBAC over a repeat cesarean delivery is an important clinical outcome and goal to examine in future research and reviews.

## Additional file

Additional file 1: **Appendix 1.** Search strategy. **Appendix 2.** Characteristics of included studies. **Appendix 3.** Methodological quality assessments of included studies. (PDF 424 kb)

## Abbreviations

ACOG: American College of Obstetrics and Gynecology
aOR: adjusted odds ratio
BMI: Body mass index
CI: Confidence interval
GA: Gestational age
GRADE: Grading of Recommendations Assessment, Development and Evaluation
MMAT: Mixed Methods Appraisal Tool
NRCT: Non-randomized clinical trial
OR: Odds ratio
RCT: Randomized clinical trial
RR: Risk ratio
SMFM: The Society for Maternal-Fetal Medicine
SOGC: The Society of Obstetricians and Gynaecologists of Canada
TOLAC: Trial of labor after cesarean
VBAC: Vaginal birth after cesarean
