# Supplementary material for: Adjunct clinical interventions that influence vaginal birth after cesarean rates: systematic review
Source: BMC Pregnancy Childbirth. 2018 Nov 21;18:452. doi: 10.1186/s12884-018-2065-x (PMC6249876; doi:10.1186/s12884-018-2065-x)
Supplement: Supplementary file 1 — Appendix 1. Search strategy. Appendix 2. Characteristics of included studies. Appendix 3. Methodological quality assessments of included studies. (PDF 424 kb) [file 12884_2018_2065_MOESM1_ESM.pdf]

Appendix 1. Search Strategy

Database: Ovid MEDLINE(R) Epub Ahead of Print, In-Process & Other Non-Indexed Citations, Ovid MEDLINE(R) Daily and Ovid MEDLINE(R) 1946 to Present
Date search conducted: 19 May 2017
Strategy:
1 Vaginal Birth after Cesarean/ (1420)
2 Trial of Labor/ (1051)
3 TOLAC*.tw,kf. (114)
4 (trial adj2 labo?r).tw,kf. (1119)
5 ((vaginal birth or vaginal delivery) adj2 c?esarean*).tw,kf. (1692)
6 VBAC*.tw,kf. (608)
7 or/1-6 [Combined MeSH & text words for VBAC] (3326)
8 exp animals/ not humans/ (4401774)
9 7 not 8 (3308)
10 limit 9 to (english or french) (3064)
11 limit 10 to yr="1985-Current" (2922)
12 remove duplicates from 11 (2792)

Database: Ovid Embase 1980 to 2017 Week 20
Date search conducted: 19 May 2017
Strategy:
1 "trial of labor"/ (848)
2 vaginal birth after cesarean/ (118)
3 TOLAC*.tw,kw. (249)
4 (trial adj2 labo?r).tw,kw. (1496)
5 ((vaginal birth or vaginal delivery) adj2 c?esarean*).tw,kw. (2244)
6 VBAC*.tw,kw. (934)
7 or/1-6 [Combined Emtree & text words for VBAC] (3687)
8 exp animal/ not human/ (4313786)
9 7 not 8 (3658)
10 limit 9 to (english or french) (3433)
11 limit 10 to yr="1985-Current" (3349)
12 remove duplicates from 11 (3287)

Database: Wiley Cochrane Library
Date search conducted: 19 May 2017
Strategy:
#1 [mh ^"Trial of Labor"] 38
#2 [mh ^"Vaginal Birth after Cesarean"] 57
#3 TOLAC*:ti,ab,kw 11
#4 (trial next/2 labo*):ti,ab,kw 286
#5 (("vaginal birth" or "vaginal delivery") next/2 (caesarean* or cesarean*)):ti,ab,kw 146
#6 VBAC*:ti,ab,kw 36
#7 {or #1-#6} 400
#8 #7 Publication Year from 1985 to 2017 389

Database: CINAHL Plus with Full Text via EBSCOhost
Date search conducted: 19 May 2017
Strategy:

#	Query	Limiters/Expanders	Results
S9	S6 NOT S7	Limiters - Published Date: 19850101-20171231; Language: English, French<br>Search modes - Find all my search terms	1,844
S8	S6 NOT S7	Search modes - Find all my search terms	1,869
S7	(MH "Animals+") NOT (MH "Human")	Search modes - Find all my search terms	65,962
S6	S1 or S2 or S3 or S4 or S5	Search modes - Find all my search terms	1,870
S5	VBAC*	Search modes - Find all my search terms	419
S4	("vaginal birth" or "vaginal delivery") N2 (caesarean* or cesarean*)	Search modes - Find all my search terms	1,641
S3	trial N2 labo#r	Search modes - Find all my search terms	429
S2	TOLAC*	Search modes - Find all my search terms	63
S1	(MH "Vaginal Birth After Cesarean")	Search modes - Find all my search terms	1,135

Database: Ovid PsycINFO 1806 to May Week 3 2017
Date search conducted: 19 May 2017
Strategy:
1 TOLAC*.ti,ab. (3)
2 (trial adj2 labo?r).ti,ab. (21)
3 ((vaginal birth or vaginal delivery) adj2 c?esarean*).ti,ab. (85)
4 VBAC*.ti,ab. (46)
5 or/1-4 [Combined subject headings & text words for VBAC] (113)
6 limit 5 to (english or french) (106)
7 limit 6 to yr="1985-Current" (104)

Database: Conference Proceedings Citation Index – Science (CPSI-S) & Conference Proceedings Citation Index- Social Science & Humanities (CPCI-SSH) --1990-present via Clarivate Analytics
Date search conducted: 2 May 2017
Strategy:
TS=(TOLAC* or "trial of labour" or "trial of labor" or "vaginal birth after caesarean" or "vaginal birth after cesarean" or "vaginal birth following caesarean" or "vaginal birth following cesarean" or VBAC*) Date: 2015-2017 [RF Note: selected 10 from 45]

Database: ProQuest Dissertations & Theses Global
Date search conducted: 2 May 2017
Strategy:
AB,TI(TOLAC* OR (trial NEAR/2 (labor or labour)) OR (("vaginal birth" OR "vaginal delivery") NEAR/2 (caesarean* OR cesarean*)) OR VBAC*)
Date: From January 01 1985 to December 31 2017 ; English only [no French in results set] (90)

Registry: ClinicalTrials.gov
URL: https://clinicaltrials.gov/
Date search conducted: 9 May 2018
Strategy:
Advanced Search >
Other terms: "vaginal birth after cesarean" OR VBAC OR TOLAC OR "trial of labor after cesarean" OR "trial of labour after cesarean" (23)

Appendix 2. Characteristics of included studies

Study;<br>Study design;<br>Country, setting;<br>Funding source	Study period;<br>Population, maternal age, parity;<br>Data source	Intervention	Comparator	TOLAC rate*	VBAC rate*	VBAC/TOLAC rate*	Conclusion relevant to VBAC
**Ayres-De-Campos 2015**<br><br>Non-concurrent cohort<br><br>Portugal, state owned & private hospitals & home births<br><br>No funding	Jan. 1, 2000-Sept. 30, 2014<br><br>Deliveries from state-owned & private hospitals, & home births (continental Portugal only)<br><br>Official government sources & national hospital discharge database	Concerted action to reduce CS rates (2010-2014):<br>• Visits to state-owned hospitals with CS rates >35%;<br>• Meetings with obstetric & midwifery staff to present data on rates, hospital comparisons, risks, financial aspects, proposed measures to reduce CS rates, promotion of VBAC;<br>• Training courses on fetal monitoring and simulation of obstetric emergencies; and,<br>• Hospital funding indexed to CS rate with negotiated hospital targets;<br><br>Number of deliveries:<br>2010: 82,734<br>2011: 77,469<br>2012: 71,093<br>2013: 63,383<br>Jan.-Sept. 2014: 51,478	No concerted action to reduce CS rates (2000-2009);<br><br>Number of deliveries:<br>2000: 103,468<br>2001: 96,921<br>2002: 96,972<br>2003: 94,045<br>2004: 91,156<br>2005: 90,356<br>2006: 87,805<br>2007: 85,067<br>2008: 85,679<br>2009: 81,750	NR	Total number of vaginal delivery episodes with a previous CS/total number of delivery episodes with a previous CS:<br><br>No concerted action vs. concerted action:<br>2000: 14,993/103,468 (14.5%);<br>2001: 13,298/96,921 (13.7%);<br>2002: 15,360/96,972 (15.8%);<br>2003: 13,890/94,045 (14.8%);<br>2004: 13,710/91,156 (15.0%);<br>2005: 13,147/90,356 (14.6%);<br>2006: 15,700/87,805 (17.9%);<br>2007: 15,431/85,067 (18.1%);<br>2008: 13,837/85,679 (16.2%);<br>2009: 13,399/81,750 (16.4%)<br>vs.<br>2010: 14,834/82,734 (17.9%);<br>2011: 17,624/77,469 (22.8%);<br>2012: 18,076/71,903 (25.1%);<br>2013: 16,365/63,383 (25.8%);<br>Jan.-Sept. 2014: 16,859/51,478 (32.8%)<br><br>16.4% (2009) to 32.8% (2014) = 99.8% increase, time trend, p<0.001	NR	A concerted action based on the transmission of information and training of healthcare professionals, together with the inclusion of CS rates as a criterion for hospital funding, was followed by a significant reduction in national CS rates, as well as an improvement in most related obstetric indicators (...in this group, VBAC increased significantly).
**Bellows 2016**<br><br>Non-concurrent cohort<br><br>US,<br>tertiary care academic hospital, approximately	Jul. 1, 2009-Dec. 31, 2013<br><br>Women who underwent TOLAC with at least 1 prior CD and a live, singleton gestation in cephalic	Post-2011 guideline (Jul. 1, 2011-Dec. 31, 2013), based on ACOG 2010 guideline):<br>• offering TOLAC to women with more than one prior CD;<br>• inducing labor with an unfavorable cervix; and,<br>• administering oxytocin, per hospital policy, to	Pre-2011 guideline (Jul. 1, 2009-Jul. 1, 2011);<br><br>450 women<br>Note: 1 (0.2%) had ≥3 prior CDs	NR	Overall VBAC rate (VBAC/VBAC+repeat CD):<br>Pre-guideline vs. Post-guideline:<br>NR (26.0%) vs. NR (33.3%)	Women with successful VBAC:<br>Pre-guideline vs. Post-guideline:<br>351/450 (78.1%) vs. 616/781 (78.9%), p=0.75	VBAC rates were unchanged (78.9% pre-guideline versus 78.1% post-guideline, p=0.75), however hospital VBAC rates increased after the guideline (26% versus 33%, p<0.0001).

4000 deliveries annually<br><br>Funding NR	presentation of ≥24 0/7 weeks of gestation<br><br>Maternal age, pre-2011 guideline vs. post-2011 guideline: median 29y (IQR 8) vs. 30y (IQR 8), p=0.00002<br><br>Parity, pre-2011 guideline vs. post-2011 guideline: median 2 (IQR 2) vs. 2 (IQR 2)	achieve Montevideo units (MVUs) of at least 200 and with same dosing regimen and upper limit as women receiving oxytocin without prior CD;<br><br>781 women<br>Note: 8 (0.1%) had ≥3 prior CDs					
**Bickell 1996**<br><br>Controlled before-after<br><br>US, hospitals (29% of 165 with active delivery services), with high, average and low cesarean rates from 8 designated Health Service Areas of New York State<br><br>Funding NR	1988 & 1993<br><br>Hospitals with active delivery services; labor & delivery records<br><br>Rural hospitals (reviewed + non-reviewed): ~25%<br><br>Teaching hospitals (reviewed + non-reviewed): ~one third<br><br>State Department hospital discharge database of labor & delivery records	Reviewed hospitals, external peer reviews by ACOG-trained team who visited hospital, interviewed key staff members and reviewed 100 labor & delivery records (audit & feedback);<br><br>1988:<br>45 hospitals;<br>mean 1430± 141.4 deliveries<br><br>1993:<br>45 hospitals;<br>mean 1503±152.8 deliveries	Non-reviewed hospitals, had obstetric service;<br><br>1988:<br>120 hospitals;<br>mean 1720±125.9 deliveries<br><br>1993:<br>120 hospitals;<br>mean 1720±119.2 deliveries	NR	1988:<br>Reviewed vs. non-reviewed hospitals:<br>mean % 10.1±1.4 vs. 12.1±0.9, NS (p>0.01)<br><br>1993:<br>Reviewed vs. non-reviewed hospitals:<br>mean % 24.8±2.0 vs. 24.8±1.1, NS (p>0.01)<br><br>Absolute reduction in rates (difference between % of VBAC in 1993 and 1988), reviewed vs. non-reviewed hospitals:<br>Mean % -14.6±1.4 (increased) vs. -12.7±1.1 (increased), NS (p>0.01)	NR	During the years of the program, VBAC rates increased by 14.6% and 12.7% (no statistical difference, however) at reviewed and non-reviewed hospitals, respectively.
**Cleary-Goldman 2005**	12-month period<br><br>Women eligible for a TOLAC delivery	Formal counseling: one-on-one formal antenatal counseling, in second and third trimesters prior to labor	No counseling: Non-participating patients eligible for a TOLAC delivery;<br><br>221 women	Counseling vs. no counseling:<br>44/95 (46.3%) vs.<br>85/221 (38.5%)	Counseling vs. no counseling:<br>26/95 (27.4%) vs.<br>70/221 (31.7%)	Counseling vs. no counseling:<br>26/44 (59.1%) vs.<br>70/85 (82.4%)	A trial of labor after previous cesarean delivery remains a reasonable option for selected and informed patients. Although the most satisfied patients were

Prospective cohort with controls<br><br>US,<br>tertiary care centre<br><br>Non-industry funded	Maternal age:<br>VBAC (26 women):<br>mean 28.34y±4.70<br><br>Parity:<br>VBAC (26 women):<br>Median (quartile 1, quartile 3):<br>2 (1,2)	and any indication for delivery;<br><br>95 women					those who succeeded at vaginal birth, most women valued the opportunity to attempt a vaginal birth regardless of outcome.
**Eden 2014**<br><br>RCT<br><br>US,<br>clinics in specified health systems in Oregon & health fairs<br><br>Non-industry funded	2005-2007<br><br>Women with only 1 prior cesarean & eligible for VBAC; ≥18y; pregnant with 1 fetus; low transverse uterine scar; read English or Spanish<br><br>Maternal age, decision aid vs. brochures:<br>mean 30.35y<br>vs.<br>31.88y, p=0.543<br><br>Prior VD, decision aid vs. brochures:<br>22.72% vs.<br>24.61%	Evidence-based, computerized decision aid:<br>program containing the pre-intervention baseline data collection screens, an interactive decision aid, and follow-up data collection screens;<br><br>66 women	Evidence-based educational ACOG brochures:<br>program containing pre-intervention baseline data collection screens, a pause after baseline questions to allow women to read two paper brochures, and follow-up data collection screens;<br><br>65 women	NR	Decision aid vs. brochures:<br>NR (41.0%) vs. NR (37.0%), p=0.724	NR	When women indicated they planned to have a VBAC at the completion of the intervention, they were more likely to have a VBAC. Women who were unsure about their birth decisions were likely to have repeat cesareans. For women in their third trimesters, the decision aid was more effective than the brochures for reducing conflict.
**Feldman 2015**<br><br>Cross-sectional<br><br>US,<br>community hospitals in California	Jan.-Dec. 2012 (delivery data);<br>Nov. 2012-Jan. 2014 (surveys with laborists)<br><br>Women with a history of CD<br><br>Hospital discharge data	Laborist hospitals:<br>hospitals employing laborists (n=43) with continuous 24/7 coverage (n=39) and part-time in-house coverage (nights & weekends, n=4);<br><br>36 hospitals that allow TOLAC;<br>2,621 women with prior CS	Non-laborist hospitals:<br>hospitals without laborists;<br><br>56 hospitals that allow TOLAC;<br>2,111 women with prior CS	Number of women with TOLAC/all women with prior CD:<br><br>All hospitals that allow TOLAC:<br>558/4,732 (11.8%)<br><br>Laborist vs. non-laborist hospital:<br>356/2,621 (9.5%; 95% CI 6.8-12.2%) vs.	Number of women with successful VBAC/all women w prior CD:<br><br>All hospitals that allow TOLAC:<br>387/4,732 (8.2%)<br><br>Laborist vs. non-laborist hospital:<br>253/2,621 (9.7%; 95% CI 7.7-11.6%) vs.	Number of women with successful VBAC/all women who attempted TOLAC:<br><br>All hospitals that allow TOLAC:<br>389/558 (69.8%)<br><br>Laborist vs. non-laborist hospital:<br>253/356 (71.0%; 67.4-74.6%) vs.	Hospitals with laborists were twice as likely to allow TOLAC. Since more women attempted, the overall VBAC rate was higher, resulting in a lower repeat cesarean rate.

Non-industry funded	for all live births in 2012; multiple gestations and preterm gestations excluded			201/2,111 (13.6%; 95% CI 11.1-16.1%), p=0.0318	137/2,111 (6.5%, 95% CI 4.4-8.6%), p=0.0302<br><br>Effect of laborists on successful VBAC for laboring women:<br>adjusted for patient- level factors:<br>OR 1.10 (95% CI 0.82-1.47), p=0.5417;<br>adjusted for patient- and adding hospital-level factors (forward selection):<br>OR 0.85 (95% CI 0.66-1.10), p=0.1901	136/201 (67.9%; 95% CI 63.2-72.5%), p=0.2943	
**Fraser 1997**<br><br>RCT<br><br>Canada, hospitals (11 Canadian; 1 US)<br><br>Non-industry funded	Apr. 1992-Nov. 1994<br><br>Women with a single previous low transverse cesarean; <28 weeks of gestation; planned to deliver in participating hospital; receiving prenatal care from participating hospital physician; sufficient knowledge of English or French to complete questionnaire<br><br>Maternal age, verbal group vs. document group: mean 31y±5 vs. 31y±5	Verbal prenatal education program:<br>pamphlet + 2 individualized contacts:<br>1) research nurse assessed woman's motivation to attempt VBAC & perceptions of attitudes of key person in her social network (spouse & treating obstetrician), informed women of consensus panel recommendation favoring VBAC and probability of success, and reassured re: pain relief options for labor; and,<br>2) 4 to 8 weeks later, research nurse + resource person (provided peer influence and support) - identify & discuss perceived barriers to VBAC including views of treating obstetrician; intervention individualized to woman's needs;<br><br>641 women:<br>Low VBAC motivation: 185/641 (28.9%);<br>High VBAC motivation: 456/641 (71.1%)	Document prenatal education program: written information (brief pamphlet) on benefits of VBAC over elective repeat CS; no contact with study personnel, encouraged to communicate with their physician with questions;<br><br>634 women	Number of women attempting VD/all women with a single previous cesarean:<br><br>All women, verbal vs. document program:<br>465/641 (72.5%) vs.<br>440/634 (69.4%);<br>Relative risk (RR) 1.1 (95% CI 1.0-1.1)<br><br>Women with low VBAC motivation, verbal vs. document program:<br>93/185 (50.3%) vs.<br>83/187 (44.4%);<br>RR 1.1 (95% CI 0.9-1.4)<br><br>Women with high VBAC motivation, verbal vs. document program:<br>372/456 (81.6%) vs.<br>357/447 (79.9%);<br>RR 1.0 (95% CI 1.0-1.1)	Number of women achieving VD/all women with a single previous cesarean:<br><br>All women, verbal vs. document program:<br>339/641 (52.9%) vs.<br>310/634 (48.9%);<br>RR 1.1 (95% CI 1.0-1.2)<br><br>Women with low VBAC motivation, verbal vs. document program:<br>63/185 (34.1%) vs.<br>54/187 (28.9%);<br>RR 1.2 (95% CI 0.9-1.6)<br><br>Women with high VBAC motivation, verbal vs. document program:<br>276/456 (60.5%) vs.<br>256/447 (57.3%);<br>RR 1.1 (95% CI 0.9-1.2)	Number of women achieving VD/all women attempting VD:<br><br>All women, verbal vs. document program:<br>339/465 (72.9%) vs.<br>310/440 (70.5%)<br><br>Women with low VBAC motivation, verbal vs. document program:<br>63/93 (67.7%) vs.<br>54/83 (65.1%)<br><br>Women with high VBAC motivation, verbal vs. document program:<br>276/372 (74.2%) vs.<br>256/357 (71.7%)	There was no evidence that an individualized prenatal education and support program, when offered to all women with previous cesarean delivery, results in a clinically significant increase in the rate of VBAC.

**Gardner 2014**<br><br>Non-concurrent cohort<br><br>Australia, metropolitan teaching hospital, approximately 2500 deliveries annually<br><br>Funding NR	2006 & May 2009-Oct. 2010<br><br>Women with a single prior cesarean section and presenting in their next pregnancy<br><br>Maternal age (n):<br><25y (29);<br>25-29y (81);<br>30-34y (142);<br>35-39y (116);<br>40+y (28)	After management strategies (2010):<br>after 2006, two combined management strategies were introduced:<br>1) management decisions for women attempting VBAC were only made by one of three 'Risk Associated Pregnancy" consultants, already on call for any high-risk obstetric patient, and;<br>2) a next birth after cesarean clinic adopted from the Western Australian model and adhering to the RCOG guidelines for women to attend at 20, 34 and 40 weeks of gestation with interval visits as per their usual model of care;<br><br>396 VBAC candidates	Before management strategies (2006):<br>prior to 2006, women having their next birth after cesarean attended routine antenatal care and received counselling for mode of birth on an ad hoc basis; women undergoing a trial of labor were managed by the on-call consultant obstetrician of the day;<br><br>Number of VBAC candidates NR	Number of women with TOLAC/number of women who were VBAC candidates:<br>164/396 (41.4%)<br><br>Number of women with trial of labor/number of women who desired a VBAC:<br>160/226 (70.8%)	Total VBAC rate for next birth after primary cesarean/all eligible women, before vs. after:<br>NR (17.2%) vs.<br>107/396 (27.0%),<br>p<0.001	Women with successful VBAC/women with TOLAC, before vs. after:<br>NR vs. 107/160 (66.9%)	In this study of women with a previous single cesarean section presenting in their next pregnancy, VBAC rates were significantly improved by introducing a dedicated next birth after cesarean antenatal clinic combined with standardized consultant labor management.
**Kosecoff 1987**<br><br>Retrospective cohort<br><br>US, acute, non-specialty, non-federal hospitals in Washington state with >150 beds<br><br>Non-industry funded	Jan. 1979-Sept. 1980 & Jul. 1981-Jun. 1982<br><br>Women with previous low transverse cesarean section<br><br>Hospital medical records	Period 3 (Jul. 1981-Jun. 1982), after conference recommendations);<br><br>1981-1982: 70 women	Period 1 (Jan. 1979-Dec. 1979), before conference recommendations &<br>Period 2 (Jan. 1980-Sept. 1980), before conference recommendations;<br><br>1979: 35 women<br>1980: 64 women	Women with TOLAC/women with previous low transverse cesarean, period 1 vs. 2 vs. 3:<br>2/35 (5.7%) vs.<br>7/64 (10.9%) vs.<br>20/70 (28.6%)<br><br>Change/month, for all time periods:<br>0.90 (0.22%), p<0.001; positive linear trend<br><br>After vs. before, adjusted:<br>2.4 (5.8%); positive linear trend	Women with vaginal delivery/ women with previous low transverse cesarean section, period 1 vs. 2 vs. 3:<br>2/35 (5.7%) vs.<br>4/64 (6.3%) vs.<br>11/70 (15.7%)<br><br>Change/month, for all time periods:<br>0.41 (0.17%), p<0.05; positive linear trend<br><br>After vs. before, adjusted:<br>2.1 (4.5%); positive linear trend	Vaginal delivery occurred/women with TOLAC, period 1 vs. 2 vs. 3:<br>2/2 (100%) vs.<br>4/7 (57.1%) vs.<br>11/20 (55.0%)	Conference recommendations may have resulted in an increased trial of labor and vaginal delivery rates in women who had had a previous transverse cesarean section. Judged by the more stringent standard of whether this represents a significant rate of change, results are not significant for either measure, although the small sample sizes in time period 1 make it difficult to detect anything short of a very large effect.
**Liu 2013**<br><br>Non-concurrent cohort	Jun. 2001-Jul. 2002 & Aug. 2005-2010<br><br>Pregnant women who delivered by cesarean section	Period 3 (Aug. 2005-2010):<br>After Global Budget System (GBS) &<br>Hospital-based self-management program (HBSM):	Period 1 (Jun. 2001-Jul. 2002):<br>Taiwan National Health Insurance Program (NHIS) implemented 1995, before GBS:	NR	Number of women with VD/women with previous CS:<br><br>Period 1 vs. 2:<br>38/800 (4.8%) vs.<br>231/1,887 (12.2%), p<0.001	NR	VBAC was affected significantly at the beginning because of incentive mechanisms such as policy implementation and encouragement, in which reimbursement of

Taiwan, tertiary hospital<br><br>Funding NR	at Chang Gung Memorial Hospital<br><br>Number of deliveries, period 1 vs. period 2 vs. period 3:<br>4,988 vs. 11,680 vs. 18,948	a strategy employing postoperative peer reviews and audits to reduce medical service costs incurred by cesarean section;<br><br>2,621 deliveries with previous CD	provided fee-for-service health care on a population basis;<br>800 deliveries with previous CD; &<br><br>Period 2 (Jul. 2002-Aug. 2005):<br>After GBS before HBSM:<br>GBS - direct and complete government funding of hospitals on a prospective basis. Results in resource allocation and cost control, including cost containment, funding certainty, easier and cheaper administration, improved coordination and planning of services, and elimination of unnecessary services;<br><br>1,887 deliveries with previous CD		Rate of improvement in period 1:<br>rate ratio 1.22 (95% CI 1.11-1.35), p<0.001;<br>Change of rate from period 1 to period 2:<br>rate ratio 0.82 (95% CI 0.74-0.90), p=0.0001;<br><br>Period 2 vs. 3:<br>231/1,887 (12.2%) vs. 298/2,621 (11.4%), p=0.3950<br>Change of rate from period 2 to period 3:<br>rate ratio 0.98 (95% CI 0.96-0.99), p=0.0003		VBAC costs would be equivalent to cesarean delivery, but it reached a plateau because of the potential risk of uterine rupture.
**Lomas 1991**<br><br>RCT, 3-arm<br><br>Canada, non-teaching hospital with ≥100 beds (minimum ≥10 obstetrical)<br><br>Non-industry funded	1988-1989 (24 months)<br><br>Women with a single previous CS, non-vertical scar, miscellaneous contraindications<br><br>Maternal age, AF vs. OLE vs. control:<br>mean 29.1y vs. 29.3y vs. 28.9y, F test=0.12, p=0.89<br><br>Parity, AF vs. OLE vs. control:<br>mean 1.16 vs. 1.13 vs. 1.15, F test=0.34, p=0.70<br><br>Hospital charts	Audit & feedback (AF, throughout 1988):<br>each obstetrics department:<br>1) establish departmentally agreed-on criteria for the use of cesarean section in cases of women with a previous cesarean section;<br>2) to have medical audits of the charts of all women with a previous cesarean section and to compare actual practice with the agreed-on criteria; and,<br>3) to hold meetings of the entire department every 3 months during 1988 for feedback and discussion of the audit results. Feedback information was prepared by the study team, the mean element in the feedback was a readily	Control (Jan. 1988):<br>a copy of the practice guideline was mailed to all who were engaged in obstetrical care (including family physicians). A brief exhortatory letter drew attention to the portion of the guideline that addressed the use of cesarean section for women with previous cesarean section, pointed out that the guideline had been endorsed by the national obstetrical specialty society, and requested that physicians implement the recommendations;<br><br>8 hospitals;<br>1,233 women with previous CS eligible for TOLAC	Women with a TOLAC/ women with previous CS eligible for TOLAC,<br>AF vs. OLE vs. control:<br>112/524 (21.4%; 95% CI 13.9-29.0) vs.<br>282/739 (38.2%; 95% CI 30.6-45.7) vs.<br>349/1,233 (28.3%; 95% CI 23.0-33.7)<br><br>Difference between OLE vs. control + AF combined:<br>+ 46%; F test=7.86, p=0.007	Women with VD/ women with previous CS eligible for TOLAC,<br>AF vs. OLE vs. control:<br>62/524 (11.8%; 95% CI 5.8-17.7) vs.<br>187/739 (25.3%; 95% CI 19.3-31.2) vs.<br>179/1,233 (14.5%; 95% CI 10.3-18.7)<br><br>Difference between OLE vs. control + AF combined:<br>+85%; F test=9.74, p=0.003	Women with vaginal birth/ women with previous CS who had TOLAC,<br>AF vs. OLE vs. control:<br>62/112 (55.4%) vs.<br>187/282 (66.3%) vs.<br>179/349 (51.3%)	Physicians with compliant opinion leaders had a trial of labor rate of 50.5% (41.6% to 59.4% CI) and a VBAC rate of 33.2 % (26.2% to 40.2% CI), 93% and 142% higher than in the comparison groups (p<0.001). Opinion leaders with educational support can generate community-wide change when they agree to be agents of change. The extent of failure with the OLE strategy can be partly explained by patient factors. Some reluctance to implement the guideline clearly remained despite the opinion leaders' efforts.

		understood tree-diagram presenting the choice points along the path to a vaginal delivery;<br><br>4 hospitals;<br>524 women with previous CS eligible for TOLAC<br><br>Opinion leader feedback (OLE):<br>all physicians engaged in obstetrical care at hospitals were mailed a questionnaire, asking them to nominate the local colleague(s) who best matched set descriptions of an educationally influential opinion leader. These 4 physicians attended a 1.5day workshop on evidence for the practice guideline's recommendations and on basic principles of behavior change. They agreed to a minimum of the following steps:<br>1) a mailing, under the physician's name & with a covering letter, of an information binder for each physician engaged in obstetrical care;<br>2) a mailing, for later inclusion in the binder, of two further detailing sheets over the first 4 months of 1988, addressing topics that the opinion leaders agreed were of concern to colleagues who might wish to consider implementing the recommendations of the practice guidelines;<br>3) to host, in the community, a meeting					

		with an expert speaker who was both knowledgeable and credible in the area of VBAC; and,<br>4) to maintain and enhance their regular formal and informal educational contacts with colleagues and to record these contacts in logbooks for the 12 months of active intervention in 1988;<br><br>4 hospitals;<br>739 women with previous CS eligible for TOLAC					
**Montgomery 2007**<br><br>RCT<br><br>UK, maternity units of hospitals in South West England & Scotland<br><br>Non-industry funded	May 2004 - August 2006<br><br>Pregnant women with one previous lower segment CS, no current obstetric problems, and delivery expected at ≥37 weeks of gestation; women of all parity were included, but their most recent delivery must have been a cesarean section<br><br>Parity, 1, decision analysis vs. information vs. usual care: 217/245 (89%) vs. 227/250 (92%) vs. 225/247 (91%);<br><br>Parity, 2, decision analysis	Decision analysis:<br>1) given information about outcomes associated with planned VD, elective CS & emergency CS;<br>2) mode of delivery was recommended based on utility assessments performed by the woman combined with probabilities of clinical outcomes within a concealed decision tree;<br><br>235 women eligible for follow-up of primary outcomes (decision conflict & mode of delivery)<br><br>Information program: women navigated through descriptions and probabilities of clinical outcomes for mother and baby associated with planned vaginal birth, elective CS & emergency CS;	Usual care: standard care given by obstetric and midwifery staff;<br><br>239 women eligible for follow-up of primary outcomes	NR	Women with VD/women eligible for follow-up of primary outcomes, decision analysis vs. information vs. usual care:<br>88/235 (37.4%) vs.<br>70/240 (29.2%) vs.<br>72/238 (30.3%)<br><br>Difference between groups, for vaginal vs. elective/emergency CS, adjusted for preferred mode of delivery at baseline, hospital & value of outcome at baseline (for decision conflict scale only):<br>Decision analysis vs. usual care:<br>aOR 1.42 (95% CI 0.94-2.14), p=0.22;<br>Information vs. usual care:<br>aOR 0.93 (95% CI 0.61-1.41), p>0.9;<br>Decision analysis vs. information:<br>aOR 1.53 (95% CI 1.01-2.30), p=0.11	NR	Both decision aids (decision analysis & information program) were associated with greater knowledge and less anxiety compared with usual care. The intervention based on decision analysis was associated with a higher proportion of women achieving vaginal birth.

	vs. information vs. usual care: 19/245 (8%) vs. 11/250 (4%) vs. 16/247 (6%);<br><br>Parity, ≥3, decision analysis vs. information vs. usual care: 7/245 (3%) vs. 10/250 (4%) vs. 6/247 (2%)	241 women eligible for follow-up of primary outcomes					
**Myers 1993**<br><br>Follow-up to non-concurrent cohort (1985-1987)<br><br>US, level 3 prenatal center<br><br>Funding NR	1985 -1991<br><br>All patients in the obstetric department<br><br>Primigravida, per year, n (%)<br>1985: 399/1697 (22.9%)<br>1986: 606/2101 (28.8%)<br>1987: 683/2301 (29.7%)<br>1988: 761/2340 (31.3%)<br>1989: 806/2688 (29.9%)<br>1990: 785/2817 (27.8%)<br>1991: 941/3218 (29.2%)<br><br>Hospital perinatal database	Post-intervention (program implemented Jan. 1, 1986):<br>1) second opinion by a board-certified obstetrician was required for all cesarean sections (not only primary CS)<br>2) Department recognized in principle that vaginal delivery was preferred for all patients who had previously undergone cesarean section<br>3) Diagnosis of dystocia was accepted as an indication for a cesarean delivery only after no progress of labor was observed for more than 2 hours of regular uterine contractions of appropriate strength<br>4) Diagnosis of fetal distress, based on monitoring of the fetal heart rate, had to be corroborated by sampling blood from fetal scalp if feasible<br>5) Vaginal delivery was recommended for all breech fetuses with the exception of those with true hyperextension of the cervical spine or macrosomia (>4300g)	Pre-intervention (1985), before the hospital initiative;<br><br>122 women with a history of CS	Women with TOLAC/women with a history of CS:<br><br>Pre-intervention:<br>1985: 55/122 (45.0%)<br>Post-intervention:<br>1986: 132/193 (68.4%)<br>1987: 233/271 (86.0%)<br>1988: 243/275 (88.3%)<br>1989: 255/279 (91.3%)<br>1990: 312/365 (85.4%)<br>1991: 374/457 (81.8%)	Women with successful VBAC/women with a history of CS<br><br>Pre-intervention:<br>1985: 29/122 (23.8%)<br>Post-intervention:<br>1986: 106/193 (54.9%)<br>1987: 162/271 (59.8%)<br>1988: 167/275 (60.1%)<br>1989: 188/279 (67.4%)<br>1990: 242/365 (66.3%)<br>1991: 291/457 (63.7%)	Women with successful VBAC/women who attempted TOLAC<br><br>Pre-intervention:<br>1985: 29/55 (52.7%)<br>Post-intervention:<br>1986: 106/132 (80.3%)<br>1987: 162/233 (69.5%)<br>1988: 167/243 (73.7%)<br>1989: 188/255 (73.7%)<br>1990: 242/312 (77.5%)<br>1991: 291/374 (77.8%)	An initiative of second opinion and stringent criteria for cesarean sections within an obstetrics department can reduce cesarean-section rates substantially without adverse effects for mother or infant.

		6) Comprehensive process of peer review was instituted to ensure adherence to the 5 guidelines;<br><br>Number of women with history of CS:<br>1986: 193<br>1987: 271<br>1988: 275<br>1989: 279<br>1990: 365<br>1991: 457					
**Pinette 2004**<br><br>Non-concurrent cohort<br><br>US, birth certificate & hospital reported data<br><br>Funding NR	1998 – 2001<br><br>Women giving birth at ≥20 weeks of gestation in the state of Maine<br><br>Hospitals, birthing center (1), home births; birth certificate data and hospital-reported data used to determine birth rates	Post-intervention (after ACOG guideline change, after Oct. 1998): ACOG revised the Practice Bulletin on VBAC delivery as follows: 'Because uterine rupture may be catastrophic, vaginal birth after Cesarean should be attempted in institutions equipped to respond to emergencies with physicians readily available to provide emergency care'. In this version, 'readily' was not well defined. In July 1999, the statement was further clarified by changing 'readily' to 'immediately'. There as the requirement of the presence of a surgeon, anesthesiologist, and operating personnel throughout the trial of labor;<br><br>Birth certificate data: number of women with previous CS:<br>1999: 1,447<br>2000: 1,548<br>2001: 1,468<br>Hospital-reported data:	Pre-intervention (before ACOG guideline, before 1998 data);<br><br>Birth certificate data:<br>1988: 1,410 women with previous CS;<br>Hospital-reported data:<br>1988: 1,386 women with previous CS	NR - Authors report attempted TOLACs not recoverable from data sources	Statewide rates of VBAC delivery<br><br>Women with successful VBAC delivery/women with previous CS<br><br>Birth certificate data:<br>Pre-intervention<br>1998: 424/1,410 (30.1%)<br>Post-intervention<br>1999: 327/1,447 (22.6%)<br>2000: 277/1,548 (17.9%)<br>2001: 193/1,468 (13.1%)<br><br>Hospital-reported data:<br>Pre-intervention<br>1998: 489/1,386 (35.3%)<br>Post-intervention<br>1999: 411/1,453 (28.3%)<br>2000: 321/1,390 (23.1%)<br>2001: 156/1,172 (13.3%)<br><br>1998 vs. 2001<br>Birth certificate data:<br>RR 2.8 (95% CI 2.5-3.2), p<0.01;<br>Hospital-reported data:<br>RR 3.5 (95% CI 3.1-4.2), p<0.01	NR	A marked decline in VBAC from 1998 to 2001 occurred after the change in ACOG vaginal birth after cesarean policy. Multiple factors have contributed to this decline, including patients refusing VBAC after counseling and inability of institutions to meet ACOG guidelines.<br><br>Many family practice physicians wrote that the most common reason for their decrease in VBAC rates was lack of back up from the obstetric service. Interestingly, 3 of 4 nurse midwives practicing home births reported an increase in HBACs in their practices since implementation of current ACOG guidelines.

		number of women with previous CS:<br>1999: 1,453<br>2000: 1,390<br>2001: 1,172					
**Russillo 2008**<br><br>Cross-sectional<br><br>Canada, secondary care urban hospital<br><br>Non-industry funded	January 1995-December 2003<br><br>Pregnant women with at least one previous CS, a birth weight of ≥500g, who delivered singletons.<br><br>Maternal age, successful VBAC by obstetricians vs. successful VBAC by family physicians:<br>mean 31.4y vs. 30.1y, p=0.002<br><br>Number of previous CS, 1, successful VBAC by obstetricians vs. successful VBAC by family physicians:<br>96.0% vs. 99.2%<br><br>Number of previous CS, 2, successful VBAC by obstetricians vs. successful VBAC by family physicians:<br>3.6% vs. 0.8%<br><br>Number of previous CS, 3, successful VBAC by	Obstetricians: take 24h in-house calls and available for emergency calls; access to emergency CS and provide support for patients in labor;<br><br>Data from 30 obstetricians;<br>3,493 women with a previous CS delivered with an obstetrician.	Family physicians: have on-call system (no in-house 24h a day); access to emergency CS and provide support for patients in labor;<br><br>Data from 13 family medicine physicians;<br>201 women with a previous CS delivered with a family physician.	Women attempting vaginal delivery among women with prior CS/women with prior CS<br><br>Obstetrician:<br>1,768/3,493 (50.6%)<br>Family physician:<br>163/201 (81.1%),<br>p<0.001	Women with VBACs/women with prior CS<br><br>Obstetrician:<br>1,136/3,493 (32.5%)<br>Family physician:<br>124/201 (61.7%)	Women with VBACs/women with TOLAC<br><br>Obstetrician:<br>1,136/1,768 (64.3%)<br>Family physician:<br>124/163 (76.1%),<br>p=0.002	More patients of family physicians than of obstetricians attempted trial of labor and had successful VBAC. Given the similarity in patient profiles, the differences in delivery outcomes may be attributable to differences in physician practice styles.

	obstetricians vs. successful VBAC by family physicians: 0.4% vs. 0.0%<br><br>Labor and delivery database from St Mary's Hospital Center						
**Sanchez-Ramos 1990**<br><br>Non-concurrent cohort<br><br>US, regional perinatal center serving almost exclusively indigent population, approximately 4500 deliveries annually<br><br>Funding NR	1986-1989<br><br>Women with one or two previous CS, with low transverse or vertical scars not extending into uterine corpus<br><br>Hospital records	Post-intervention (after Jul. 1, 1987): new guidelines regarding intrapartum management of women with prior cesarean sections were introduced. At weekly conferences, departmental resident and obstetric faculty physicians reviewed each cesarean section and focused on indications for abdominal delivery;<br><br>Women with previous CS:<br>1988: 525<br>1989: 580	Pre-intervention (before Jul. 1, 1987): no guideline change;<br><br>Women with previous CS:<br>1986: 438<br>1987: 461	Women with TOLAC/ women with prior cesarean section:<br><br>Pre-intervention:<br>1986: 139/438 (31.7%)<br>1987: 193/461 (41.9%)<br>Post-intervention:<br>1988: 402/525 (76.5%)<br>1989: 487/580 (84.0%)<br><br>Difference (1986-1989): 52.2%, p<0.0001	Women with successful VBAC/women with prior CS:<br><br>Pre-intervention:<br>1986: 90/438 (20.5%)<br>1987: 142/461 (30.8%)<br>Post-intervention:<br>1988: 342/525 (65.1%)<br>1989: 403/580 (69.5%)<br><br>Difference (1986-1989): 48.9%, p<0.0001	Women with subsequent vaginal birth/women with TOLAC:<br><br>Pre-intervention:<br>1986: 90/139 (64.7%)<br>1987: 142/193 (73.6%)<br>Post-intervention:<br>1988: 342/402 (85.1%)<br>1989: 403/487 (82.8%)<br><br>Difference (1986-1989): 18.0%, p<0.0001	From 1986 to 1989 the proportion of patients with prior cesarean deliveries who underwent a trial of labor increased from 32% to 84% (p<0.0001). The proportion of women undergoing a trial of labor who had a subsequent vaginal births increased from 65% to 83% (p<0.0001). Among all women who delivered after a previous cesarean section, subsequent vaginal birth increased from 20.5% to 69.4% (p<0.0001). We support maintenance of selective criteria for vaginal delivery of breech presentation fetuses. The success in lowering CS rates is largely attributable to the centralized approach to intrapartum decision making.
**Santerre 1996**<br><br>Non-concurrent cohort<br><br>US, hospitals in Massachusetts<br><br>Funding NR	1985-1993<br><br>Births for which the mother had previously given birth by cesarean section<br><br>55 Massachusetts hospitals; 47,480 deliveries	Post-intervention (after ACOG practice guideline, issued Oct. 1988): guideline stating that a prior cesarean section is no longer a reason for performing a repeat C-section;<br><br>Number of women/deliveries NR	Pre-intervention (before 1987-1988): prior to issuance of physician guideline;<br><br>Number of women/deliveries NR	NR	VBAC rate in the US (data for Massachusetts hospitals NR)<br><br>Pre-intervention:<br>1985: 6.6%<br>1986: 8.5%<br>1987: 9.8%<br>1988: 12.6%<br>Post-intervention:<br>1989: 18.5%<br>1990: 20.4%<br>1991: 24.2%<br>1992: 25.1%<br>1993: 25.4%	NR	The study suggests that practice guidelines do sometimes work. The VBAC rate at the typical hospital in Massachusetts increased by about 5.6 percentage points as a result of the ACOG guideline. The information dissemination role of the popular press may provide the reason why the ACOG guideline influenced the practice of VBACs.

**Studnicki 1997**<br>Non-concurrent cohort<br><br>US, non-federal acute care provider hospitals<br><br>Funding NR	1990-1993<br><br>Only discharge codes representing CS and vaginal births were included in the study<br><br>Maternal age, early adopter vs. late adopter vs. nonqualified:<br><18y: 5.7% vs. 5.7% vs. 2.5%;<br>18-35y: 87.3% vs. 86.5% vs. 88.8%;<br>>35y: 7.0% vs. 7.8% vs. 8.7%<br><br>Magnetic tapes containing hospital discharge data from non-federal acute care provider hospitals	Post-intervention (1993): after guideline implementation, the section on labor diagnosis refers to indicators and common diagnoses associated with cesarean deliveries with an emphasis on maternal and fetal limitations associated with vaginal birth after a previous cesarean delivery;<br><br>Women with prior CS:<br>1993: 23,142	Pre-intervention (1990-1992): prior to implementation of practice parameters to be followed by physicians in defined hospitals when performing cesarean deliveries;<br><br>Women with prior CS:<br>1990: 22,091<br>1991: 21,641<br>1992: 22,970	NR	Vaginal births with prior cesarean:<br><br>Pre-intervention:<br>1990: 4,816/22,091 (21.8%)<br>1991: 5,540/21,641 (25.6%)<br>1992: 6,133/22,970 (26.7%)<br>Post-intervention<br>1993: 7,151/23,142 (30.9%)	NR	Mere dissemination of practice guidelines by a state agency may not achieve either the magnitude or the specificity of the results desired without an explicit and thorough guideline implementation program.
**White 2016**<br><br>Non-concurrent cohort<br><br>UK, tertiary teaching hospital<br><br>Non-industry funded	2008 (pre) & 2011 (post)<br><br>Women with one previous cesarean who received antenatal and intrapartum care at the hospital during 2008 and 2011<br><br>Maternal age, 2008 vs. 2011: mean 30.67y±4.89 vs. 30.86y±4.95	Post-intervention (2011): women who received midwife-led antenatal care; had all of their care from a midwife, including support with making their mode of birth choice;<br><br>196 women	Pre-intervention (2008): women who received traditional obstetrician-led antenatal care; attended up to 3 appointments with a hospital doctor under the auspices of their consultant obstetrician and received the rest of their care from a community midwife. Hospital doctor took the main role in supporting women to make their mode of birth choice;<br><br>209 women	Women who attempted VBAC/women with previous CS,<br>pre-intervention vs. post-intervention:<br>143/209 (68.4%) vs.<br>153/196 (78.1%)	Women with actual VBAC/women with previous CS,<br>pre-intervention vs. post-intervention:<br>98/209 (46.9%) vs.<br>120/196 (61.2%);<br><br>Difference in actual VBAC: aOR 1.79 (95% CI 1.17-2.75), p<0.05<br><br>Women with spontaneous VBAC/women with previous CS, pre-intervention vs. post-intervention:<br>67/209 (32.1%) vs.<br>85/196 (43.4%)	Women with actual VBAC/women who attempted VBAC,<br>pre-intervention vs. post-intervention:<br>98/143 (68.5%) vs.<br>120/153 (78.4%)<br><br>Difference in actual VBAC of women who attempted VBAC: OR 1.67 (95% CI 0.99-2.82), NS (p>0.05)	Implementation of midwife-led antenatal care has been shown to be associated with increased intended and actual VBAC rates, and reduced unscheduled antenatal care by way of the delivery suite and inpatient admission, with similar safety outcomes.

	Hospital obstetric database of women who delivered at the hospital in 2008 and 2011.				Difference in spontaneous VBAC:<br>OR 1.62 (95% CI 1.08-2.43)		
**Wong 2014**<br><br>Prospective cohort<br><br>UK, District general hospital in South East England<br><br>Funding NR	12-month period commencing January 1, 2012<br><br>Women with one previous lower segment cesarean section without contraindications for a VBAC	Attended one-stop obstetrician-led cesarean education and antenatal sessions (OCEANS): 1-hour discussion group where 5 to 15 women who have had one previous CS are invited to attend. Women were given written information about the risks and benefits of VBAC and ERCS, a consultant obstetrician went through the info, then facilitated a discussion where women could discuss their concerns and aspirations for their pregnancy and delivery;<br><br>188 women	Did not attend OCEANS (one-stop obstetrician-led cesarean education and antenatal sessions)- either cancelled their appointment or did not keep their appointment; normal care;<br><br>78 women (20 cancelled appointment, 58 did not keep appointment)	Women who attempted VBAC/ women with previous CS, attended OCEANS vs. did not attend OCEANS:<br>108/188 (57.4%) vs.<br>33/78 (42.3%),<br>p=0.02	Women with vaginal delivery/women with previous CS,<br>attended OCEANS vs. did not attend OCEANS:<br>59/188 (31.4%) vs.<br>20/78 (25.6%)	Women who had vaginal delivery/women who attempted VBAC,<br>attended OCEANS vs. did not attend OCEANS:<br>59/108 (54.6%) vs.<br>20/33 (60.6%),<br>p=0.69 (Table 2), reported in study abstract as 56% vs. 61%, p=0.55	The rate of successful vaginal delivery in women who attempted VBAC was 79/141 (55%), and this was not influenced by whether they attended a dedicated obstetrician-led clinic or not.
**Yee 2017**<br><br>Retrospective cohort<br><br>US, large teaching hospital<br><br>Funding NR	January 2008-June 2013<br><br>Women 18 years or older with one prior low transverse cesarean delivery and a term, cephalic singleton gestation, and no prior vaginal delivery.<br><br>Maternal age, night float vs. traditional call: mean 34.1y±4.7 vs. 33.8y±4.5, p=0.35	Night float call: Those who practiced in a group where the one-call obstetrician provided hospital care for several nights sequentially without daytime officer or other clinical responsibilities; those whose only clinical responsibility was for hospitalized patients in either a day or night shift, shifts were followed by time for sleep prior to a subsequent shift;<br><br>556 women	Traditional call: Physicians performed daytime clinical responsibilities followed by nighttime call (either home or in hospital) with possible subsequent partial or full-day clinical responsibilities the next day;<br><br>946 women	Women who had TOLAC/women with prior CD, night float call vs. traditional call:<br>184/556 (33.1%) vs.<br>156/946 (16.5%)<br><br>OR 2.50 (95% CI 1.96-3.20), p<0.001, unadjusted<br><br>aOR 2.64 (95% CI 1.65-4.25), p<0.001, adjusted for BMI, GA, and physician	Women who had VBAC/women with prior CS,<br>night float call vs. traditional call:<br>104/556 (18.7%) vs.<br>88/946 (9.3%)<br><br>OR 2.24 (95% CI 1.65-3.05), p<0.001, unadjusted<br><br>aOR 2.17 (95% CI 1.36-3.45), p<0.001, adjusted for BMI, GA, and physician	Women who had VBAC/ women who had TOLAC,<br>night float call vs. traditional call:<br>104/184 (56.5%) vs.<br>88/156 (56.4%)<br><br>OR 1.00 (95% CI 0.65-1.55), p=0.98, unadjusted<br><br>OR 0.96 (95% CI 0.57-1.62), p=0.98, adjusted for BMI, GA, and physician	In summary, we identified that in a single, large teaching hospital, women who were eligible for a trial of labor after cesarean were more likely to undergo a trial of labor after cesarean if delivered by physicians in a night float call system, and the increased odds of experiencing a trial of labor after cesarean translated to an increased odds of vaginal birth after cesarean.

	Data from electronic medical records.						
**Zhang 2016**<br><br>RCT<br><br>China, hospital obstetric department<br><br>No funding	May 2013 – November 2014<br><br>Women in labor who had a history of previous cesarean section and received vaginal birth in obstetrical department; willingness to participate in the study and have a vaginal birth, without indications of abnormal delivery such as multiple pregnancies, high risk pregnancy, placenta or amniotic fluid problems, without having mental diseases or problems in which the mother cannot communicate with others, and all the participants had a history of previous cesarean section.<br><br>Maternal age: range 25-40y, p>0.05	Continuing midwifery care: midwife provided care during the antenatal, labor and birth, and postnatal periods according to the National Midwifery Guidelines.<br><br>N=48 women	Standard maternity care: antenatal staff, including midwives or obstetricians, provided antenatal care. Staff in birth unit provided labor and birth care and midwives in postnatal ward provided postnatal care.<br><br>N= 48 women	NR	Women with successful VBAC/women with previous CS,<br>continuing midwifery care vs. standard maternity care:<br>42/48 (87.5%) vs.<br>32/48 (66.7%),<br>p<0.05	NR	Although VBAC rates are related to many factors, such as the structure of the maternity care system, the cooperation between midwives and obstetricians and the sociocultural influence, the continuous presence of a midwife in all the stages of labor will promote a woman's body to generate endogenous analgesic or endorphin.
**Zweifler 2006**	1996-2002	Post-intervention (2000-2002): After the ACOG VBAC guideline revision.	Pre-intervention (1996-1999): Before the ACOG VBAC guideline revision	All live births with attempted VBAC/all live births with previous CS,	All live births with successful VBAC/all live births with previous CS,	All live births with successful VBAC/all live births with attempted VBAC, pre-	Neonatal and maternal mortality rates did not improve despite increasing

Non-concurrent cohort<br><br>US, California Department of Health Services Birth Statistical Master Files<br><br>Funding NR	Total births in California: 3,545,518; previous CS: 386,232<br><br>Maternal age of all women with a previous CS who attempt VBAC:<br>Pre-intervention:<br><20y: 26.6%<br>20-29y: 25.2%<br>30-39y: 23.3%<br>40-49y: 19.3%<br>Post-intervention:<br><20y: 11.7%<br>20-29y: 13.9%<br>30-39y: 13.4%<br>40-49y: 11.2%<br><br>California Birth Statistical Master Files	Called for the immediate availability of cesarean section capability.		pre-intervention vs. post-intervention:<br>50,670/NR (%NR) vs.<br>23,573/NR (%NR)<br><br>Women who attempted VBAC (1996-2002)/women with previous CS (1996-2002):<br>74,243/386,232 (19.2%)<br><br>Women who attempted VBAC/women with previous CS, pre-intervention vs. post-intervention:<br>NR (24%) vs.<br>NR (13.5%)<br><br>Difference pre- vs. post-ACOG guideline revision:<br>44% decrease, p<0.001	pre-intervention vs. post-intervention:<br>41,961/NR (%NR) vs.<br>19,723/NR (%NR)<br><br>Women with successful VBAC (1996-2002)/women with previous CS (1996-2002):<br>61,684/386,232 (16.0%)	intervention vs. post-intervention:<br>41,961/50,670 (82.8%) vs.<br>19,273/23,573 (81.8%)<br><br>All live births with successful VBAC (1996-2002)/all live births with attempted VBAC (1996-2002), rural vs. urban:<br>NR (79.5%) vs.<br>NR (83.3%)	rates of repeat cesarean delivery during the years after the ACOG 1999 VBAC guideline revision. Women with infants weighing ≥1500g encountered similar neonatal and maternal mortality rates with VBAC or repeat cesarean delivery.

TOLAC: trial of labor after cesarean; VBAC: vaginal birth after cesarean; CS: cesarean section; NR: not reported; vs.: versus; US: United States; CD: cesarean delivery; y: year(s); IQR: interquartile range; ACOG: American College of Obstetricians and Gynecologists; MVU: Montevideo unit; NS: not significant; CI: confidence interval; RCT: randomized controlled trial; VD: vaginal delivery; OR: odds ratio; RR: relative risk; RCOG: Royal College of Obstetricians and Gynaecologists; GBS: Global Budget System; HBSM: hospital-based self-management; NHIS: National Health Insurance System; AF: audit and feedback; OLE: opinion leader education; UK: United Kingdom; aOR: adjusted odds ratio; HBAC: home birth after cesarean; OCEANS: obstetrician-led cesarean education and antenatal session; ERCS: elective repeat cesarean section; BMI: body mass index; GA: gestational age

* Results of statistical tests or summary statistics were extracted whenever these were reported within studies

Appendix 3. Methodological quality of included studies

**MMAT* criteria**	**Author's judgment**	**Support for judgment**
**Ayres-de-Campos, 2015 (non-concurrent cohort)**		
Are there clear qualitative and quantitative research questions (or objectives), or a clear mixed methods question (or objective)?	Yes	To evaluate national cesarean section rates and other obstetric indicators after a concerted action to reduce cesarean section.
Do the collected data allow address the research question (objective)? E.g. consider whether the follow-up period is long enough for the outcome to occur (for longitudinal studies or study components).	Yes	Reported delivery rates from government sources and hospital discharge database.
3.1 Are participants (organizations) recruited in a way that minimizes selection bias?	Yes	All births from state-owned hospitals, private hospitals and home births were selected.
3.2 Are measurements appropriate (clear origin, or validity known, or standard instrument; and absence of contamination between groups when appropriate) regarding the exposure/intervention and outcomes?	Yes	Outcome is reported as VBAC rates, concerted action during 2010 and early 2011.
3.3 In the groups being compared (exposed vs non-exposed; with intervention vs without; cases vs controls), are the participants comparable, or do researchers take into account (control for) the difference between these groups?	No	No explicit statement, no comparison table for groups.
3.4 Are there complete outcome data (80% or above), and, when applicable, an acceptable response rate (60% or above), or an acceptable follow-up rate for cohort studies (depending on the duration of follow-up)?	Yes	Appears to report all delivery outcomes, complete data.
Overall quality score	☆☆☆ (75%)	
**Bickell, 1996 (controlled before-after)**		
Are there clear qualitative and quantitative research questions (or objectives), or a clear mixed methods question (or objective)?	Yes	To assess the effectiveness of a joint-specialty society and health department peer-review program to reduce cesarean section rates.
Do the collected data allow address the research question (objective)? E.g. consider whether the follow-up period is long enough for the outcome to occur (for longitudinal studies or study components).	Yes	Data from before and after intervention reported delivery rates.
3.1 Are participants (organizations) recruited in a way that minimizes selection bias?	Yes	Reviewed and non-reviewed hospitals selected from designated Health Service Areas of New York state. Participants identified by computer randomized number generator.

3.2 Are measurements appropriate (clear origin, or validity known, or standard instrument; and absence of contamination between groups when appropriate) regarding the exposure/intervention and outcomes?	Yes	Deliveries and cesarean section rates reported, examined before and after intervention.
3.3 In the groups being compared (exposed vs non-exposed; with intervention vs without; cases vs controls), are the participants comparable, or do researchers take into account (control for) the difference between these groups?	Yes	Groups are comparable, non-significant differences.
3.4 Are there complete outcome data (80% or above), and, when applicable, an acceptable response rate (60% or above), or an acceptable follow-up rate for cohort studies (depending on the duration of follow-up)?	Yes	Appears to report all delivery outcomes, complete data.
Overall quality score	☆☆☆☆ (100%)	
**Bellows, 2016**<br>**(non-concurrent cohort)**		
Are there clear qualitative and quantitative research questions (or objectives), or a clear mixed methods question (or objective)?	Yes	To evaluate maternal-neonatal morbidity after TOLAC after ACOG guideline change.
Do the collected data allow address the research question (objective)? E.g. consider whether the follow-up period is long enough for the outcome to occur (for longitudinal studies or study components).	Yes	Delivery outcomes reported from medical records before and after guideline implementation.
3.1 Are participants (organizations) recruited in a way that minimizes selection bias?	Yes	All women attempting TOLAC at hospital were included.
3.2 Are measurements appropriate (clear origin, or validity known, or standard instrument; and absence of contamination between groups when appropriate) regarding the exposure/intervention and outcomes?	Yes	TOLAC & VBAC success rates defined and reported; pre- & post-intervention times stated and appropriate.
3.3 In the groups being compared (exposed vs non-exposed; with intervention vs without; cases vs controls), are the participants comparable, or do researchers take into account (control for) the difference between these groups?	Yes	Demographic info comparing women before vs. after guideline; controlled for confounders in multi-variate analysis by adjusting for differences in groups.
3.4 Are there complete outcome data (80% or above), and, when applicable, an acceptable response rate (60% or above), or an acceptable follow-up rate for cohort studies (depending on the duration of follow-up)?	Yes	Appears to report all delivery outcomes, complete data.
Overall quality score	☆ ☆ ☆ ☆ (100%)	
**Cleary-Goldman, 2005**<br>**(prospective cohort with controls)**		
Are there clear qualitative and quantitative research questions (or objectives), or a clear mixed methods question (or objective)?	Yes	To determine patient satisfaction with delivery experience, of those enrolled in formal VBAC educational program.

Do the collected data allow address the research question (objective)? E.g. consider whether the follow-up period is long enough for the outcome to occur (for longitudinal studies or study components).	Yes	Delivery outcomes reported, and a survey on patients' satisfaction with primary cesarean section delivery was completed.
3.1 Are participants (organizations) recruited in a way that minimizes selection bias?	No	Women chose to participate in intervention (VBAC counselling). Used data of all eligible patients for comparison.
3.2 Are measurements appropriate (clear origin, or validity known, or standard instrument; and absence of contamination between groups when appropriate) regarding the exposure/intervention and outcomes?	Yes	12-month study period, delivery outcomes reported.
3.3 In the groups being compared (exposed vs non-exposed; with intervention vs without; cases vs controls), are the participants comparable, or do researchers take into account (control for) the difference between these groups?	No	No comparison of groups.
3.4 Are there complete outcome data (80% or above), and, when applicable, an acceptable response rate (60% or above), or an acceptable follow-up rate for cohort studies (depending on the duration of follow-up)?	Yes	Appears to report all delivery outcomes, complete data.
Overall quality score	☆ ☆ (50%)	
**Eden, 2014** **(RCT)**		
Are there clear qualitative and quantitative research questions (or objectives), or a clear mixed methods question (or objective)?	Yes	To evaluate tools to help women with prior cesarean section make informed decisions about trial of labor.
Do the collected data allow address the research question (objective)? E.g. consider whether the follow-up period is long enough for the outcome to occur (for longitudinal studies or study components).	Yes	Reported delivery outcomes, with pre-intervention baseline data and follow-up data.
2.1 Is there a clear description of the randomization (or an appropriate sequence generation)?	Yes	Secured randomization database used. Women randomized in blocks based on language.
2.2 Is there a clear description of the allocation concealment (or blinding when applicable)?	No	Research assistant appears to be unblinded (loaded the decision aid on the computer, distributed the paper brochures), and performed data extraction from the computers after women used decision aid.
2.3 Are there complete outcome data (80% or above)?	No	Delivery route information for 92/131 (70%) of women; not complete data.
2.4 Is there low withdrawal/drop-out (below 20%)?	Yes	3/134 (2%) did not appear for session; low withdrawal rate.

Overall quality score	☆☆ (50%)	
**Feldman, 2015**<br>**(cross-sectional)**		
Are there clear qualitative and quantitative research questions (or objectives), or a clear mixed methods question (or objective)?	Yes	To determine impact of laborist staffing model on cesarean section rates and maternal morbidity.
Do the collected data allow address the research question (objective)? E.g. consider whether the follow-up period is long enough for the outcome to occur (for longitudinal studies or study components).	Yes	Cross-sectional look at births, from nurse managers & state hospital discharge data.
3.1 Are participants (organizations) recruited in a way that minimizes selection bias?	Yes	Data from all women with live births in hospitals with labor and delivery units in 2012.
3.2 Are measurements appropriate (clear origin, or validity known, or standard instrument; and absence of contamination between groups when appropriate) regarding the exposure/intervention and outcomes?	Yes	Surveys completed November 2012-January 2014. Surveys were validated, reported VBAC rate and birth outcome rates.
3.3 In the groups being compared (exposed vs non-exposed; with intervention vs without; cases vs controls), are the participants comparable, or do researchers take into account (control for) the difference between these groups?	Yes	Used multiple logistic regression models to account for patient and hospital level factors in outcomes. Acknowledged differences in laborist vs. non-laborist hospitals.
3.4 Are there complete outcome data (80% or above), and, when applicable, an acceptable response rate (60% or above), or an acceptable follow-up rate for cohort studies (depending on the duration of follow-up)?	Yes	Appears to report all delivery outcomes, complete data.
Overall quality score	☆ ☆ ☆ ☆ (100%)	
**Fraser, 1997**<br>**(RCT)**		
Are there clear qualitative and quantitative research questions (or objectives), or a clear mixed methods question (or objective)?	Yes	To assess if prenatal education promoting VBAC increases VBAC rates.
Do the collected data allow address the research question (objective)? E.g. consider whether the follow-up period is long enough for the outcome to occur (for longitudinal studies or study components).	Yes	Reported VBAC rates. Questionnaire to women 12h-72h post-partum & hospital chart info after discharge.
2.1 Is there a clear description of the randomization (or an appropriate sequence generation)?	Yes	Performed through a centralized telephone answering service, blocked and stratified by hospital and women's motivation to attempt vaginal delivery.
2.2 Is there a clear description of the allocation concealment (or blinding when applicable)?	Unsure	No description of allocation concealment/blinding, only that women were allocated to one of two groups.

2.3 Are there complete outcome data (80% or above)?	Yes	13/1301 (1%) women were lost to follow-up; complete outcome data.
2.4 Is there low withdrawal/drop-out (below 20%)?	Yes	1/1301 (<1%) dropped out originally; low withdrawal rate.
Overall quality score	☆ ☆ ☆ (75%)	
**Gardner, 2014 (non-concurrent cohort)**		
Are there clear qualitative and quantitative research questions (or objectives), or a clear mixed methods question (or objective)?	Yes	To determine the combined effect of two management strategies on VBAC.
Do the collected data allow address the research question (objective)? E.g. consider whether the follow-up period is long enough for the outcome to occur (for longitudinal studies or study components).	Yes	Reported VBAC rates, study conducted from May 2009 to October 2010.
3.1 Are participants (organizations) recruited in a way that minimizes selection bias?	Yes	All women with single prior cesarean section eligible for VBAC were invited.
3.2 Are measurements appropriate (clear origin, or validity known, or standard instrument; and absence of contamination between groups when appropriate) regarding the exposure/intervention and outcomes?	No	VBAC rate (pre- & post-) was not defined; VBAC rate prior to 2006 compared with period well after intervention (2009-2010).
3.3 In the groups being compared (exposed vs non-exposed; with intervention vs without; cases vs controls), are the participants comparable, or do researchers take into account (control for) the difference between these groups?	No	No note of any comparison between groups. Commented on demographic and past birth characteristics on desire for VBAC, but did not consider influence on VBAC rates or account for these in the analysis.
3.4 Are there complete outcome data (80% or above), and, when applicable, an acceptable response rate (60% or above), or an acceptable follow-up rate for cohort studies (depending on the duration of follow-up)?	Yes	1/396 (<1%) lost to follow-up; complete outcome data.
Overall quality score	☆ ☆ (50%)	
**Kosecoff, 1987 (non-concurrent cohort)**		
Are there clear qualitative and quantitative research questions (or objectives), or a clear mixed methods question (or objective)?	Yes	To assess the effectiveness of National Institute of Health consensus development program on practice of physicians
Do the collected data allow address the research question (objective)? E.g. consider whether the follow-up period is long enough for the outcome to occur (for longitudinal studies or study components).	Yes	Medical records from pre- and post-conference. Reported delivery outcomes.
3.1 Are participants (organizations) recruited in a way that minimizes selection bias?	Yes	Medical records of all acute, non-specialty nonfederal hospitals in state. Within a hospital, each patient had equal chance of being selected.

3.2 Are measurements appropriate (clear origin, or validity known, or standard instrument; and absence of contamination between groups when appropriate) regarding the exposure/intervention and outcomes?	Yes	Time period range clear, conference October 1980, delivery outcomes reported.
3.3 In the groups being compared (exposed vs non-exposed; with intervention vs without; cases vs controls), are the participants comparable, or do researchers take into account (control for) the difference between these groups?	No	No comparison of any groups. Smaller sample size in time period 1 vs. 2 & 3.
3.4 Are there complete outcome data (80% or above), and, when applicable, an acceptable response rate (60% or above), or an acceptable follow-up rate for cohort studies (depending on the duration of follow-up)?	Unsure	Records missing for outcomes (VBAC and non-VBAC included), unclear which are VBAC.
Overall quality score	☆ ☆ (50%)	
**Liu, 2013**<br>**(non-concurrent cohort)**		
Are there clear qualitative and quantitative research questions (or objectives), or a clear mixed methods question (or objective)?	Yes	To examine the impact of different national health policies on cesarean section rates at tertiary hospital.
Do the collected data allow address the research question (objective)? E.g. consider whether the follow-up period is long enough for the outcome to occur (for longitudinal studies or study components).	Yes	Delivery outcomes reported; cesarean section and VBAC rates pre- and post-intervention.
3.1 Are participants (organizations) recruited in a way that minimizes selection bias?	Yes	All deliveries by cesarean section from June 2001-August 2010 were assessed.
3.2 Are measurements appropriate (clear origin, or validity known, or standard instrument; and absence of contamination between groups when appropriate) regarding the exposure/intervention and outcomes?	Yes	Delivery rates reported. Implemented programs in July 2002 and August 2005, supported by health policy.
3.3 In the groups being compared (exposed vs non-exposed; with intervention vs without; cases vs controls), are the participants comparable, or do researchers take into account (control for) the difference between these groups?	No	Mentions cesarean section rate changes may be due to cultural and practical factors, but do not directly compare demographics between groups or account for any difference in analysis.
3.4 Are there complete outcome data (80% or above), and, when applicable, an acceptable response rate (60% or above), or an acceptable follow-up rate for cohort studies (depending on the duration of follow-up)?	Yes	Appears to report all delivery outcomes, complete data.
Overall quality score	☆ ☆ ☆ (75%)	
**Lomas, 1991**<br>**(RCT, 3-arm)**		

Are there clear qualitative and quantitative research questions (or objectives), or a clear mixed methods question (or objective)?	Yes	To evaluate whether opinion leaders vs audit & feedback lead to increases in rates of trial of labor and VBAC.
Do the collected data allow address the research question (objective)? E.g. consider whether the follow-up period is long enough for the outcome to occur (for longitudinal studies or study components).	Yes	Delivery outcomes reported from medical charts audited.
2.1 Is there a clear description of the randomization (or an appropriate sequence generation)?	Yes	Randomly selected counties, and randomly selected hospital from there.
2.2 Is there a clear description of the allocation concealment (or blinding when applicable)?	No	No clear description of allocation concealment or blinding.
2.3 Are there complete outcome data (80% or above)?	Yes	Despite 72% response rate to survey, VBAC rates were reported completely.
2.4 Is there low withdrawal/drop-out (below 20%)?	Unsure	Did not report withdrawals or losses to follow-up.
Overall quality score	☆ ☆ (50%)	
**Montgomery, 2007**<br>**(RCT)**		
Are there clear qualitative and quantitative research questions (or objectives), or a clear mixed methods question (or objective)?	Yes	To determine the effects of two computer-based decision aids on mode of delivery and decisional conflict.
Do the collected data allow address the research question (objective)? E.g. consider whether the follow-up period is long enough for the outcome to occur (for longitudinal studies or study components).	Yes	Reported mode of delivery and outcomes.
2.1 Is there a clear description of the randomization (or an appropriate sequence generation)?	Yes	Randomized women by computer sequence to 1 of 3 groups, stratified by maternity unit and preferred mode of delivery at baseline; randomly permuted.
2.2 Is there a clear description of the allocation concealment (or blinding when applicable)?	Yes	Member of staff with no other involvement in the trial performed the allocation.
2.3 Are there complete outcome data (80% or above)?	Yes	Mode of delivery data 713/742 (96%); complete outcome data.
2.4 Is there low withdrawal/drop-out (below 20%)?	Yes	5/247 (2%), 6/250 (2%) & 3/245 (1%) withdrew after randomization in groups 1, 2, and 3 respectively. Low withdrawal rate.
Overall quality score	☆ ☆ ☆ ☆ (100%)	
**Myers, 1993**<br>**(Follow-up to non-concurrent cohort [1985-1987])**		
Are there clear qualitative and quantitative research questions (or objectives), or a clear mixed methods question (or objective)?	Yes	To implement a hospital initiative to reduce cesarean section rate.

Do the collected data allow address the research question (objective)? E.g. consider whether the follow-up period is long enough for the outcome to occur (for longitudinal studies or study components).	Yes	Delivery outcomes reported from perinatal database.
3.1 Are participants (organizations) recruited in a way that minimizes selection bias?	Unsure	Not clear if initiative is implemented towards patients of all private physicians who voluntary participate.
3.2 Are measurements appropriate (clear origin, or validity known, or standard instrument; and absence of contamination between groups when appropriate) regarding the exposure/intervention and outcomes?	Yes	Delivery rates used, initiative effective January 1, 1986.
3.3 In the groups being compared (exposed vs non-exposed; with intervention vs without; cases vs controls), are the participants comparable, or do researchers take into account (control for) the difference between these groups?	Unsure	Not clear about baseline characteristics, or comparability between the years.
3.4 Are there complete outcome data (80% or above), and, when applicable, an acceptable response rate (60% or above), or an acceptable follow-up rate for cohort studies (depending on the duration of follow-up)?	Yes	Appears to report all delivery outcomes, complete data.
Overall quality score	☆ ☆ (50%)	
**Pinette, 2004**<br>**(non-concurrent cohort)**		
Are there clear qualitative and quantitative research questions (or objectives), or a clear mixed methods question (or objective)?	Yes	To evaluate the effect a more restrictive national trial of labor policy has on VBAC rates and delivery rates.
Do the collected data allow address the research question (objective)? E.g. consider whether the follow-up period is long enough for the outcome to occur (for longitudinal studies or study components).	Yes	Delivery rates reported. Pre-exposure and post-exposure time frames used.
3.1 Are participants (organizations) recruited in a way that minimizes selection bias?	Yes	All women giving birth at or more than 20 weeks of gestation, from database.
3.2 Are measurements appropriate (clear origin, or validity known, or standard instrument; and absence of contamination between groups when appropriate) regarding the exposure/intervention and outcomes?	Yes	ACOG guidelines adapted October 1998 & July 1999; overall birth rates, cesarean section and VBAC rates measured.
3.3 In the groups being compared (exposed vs non-exposed; with intervention vs without; cases vs controls), are the participants comparable, or do researchers take into account (control for) the difference between these groups?	No	Difference in reported rates in birth-certificate & hospital data not addressed. Does not compare demographics between groups.

3.4 Are there complete outcome data (80% or above), and, when applicable, an acceptable response rate (60% or above), or an acceptable follow-up rate for cohort studies (depending on the duration of follow-up)?	Yes	Appears to report all delivery outcomes, complete data.
Overall quality score	☆ ☆ ☆ (75%)	
**Russillo, 2008 (cross-sectional)**		
Are there clear qualitative and quantitative research questions (or objectives), or a clear mixed methods question (or objective)?	Yes	To determine differences between family physicians and OBGYN in trial of labor attempts, VBAC success
Do the collected data allow address the research question (objective)? E.g. consider whether the follow-up period is long enough for the outcome to occur (for longitudinal studies or study components).	Yes	Delivery rates reported, labor & delivery hospital database used.
3.1 Are participants (organizations) recruited in a way that minimizes selection bias?	Yes	All pregnant women with previous cesarean section and current singleton pregnancy were included.
3.2 Are measurements appropriate (clear origin, or validity known, or standard instrument; and absence of contamination between groups when appropriate) regarding the exposure/intervention and outcomes?	Yes	Used maternal and neonatal data from database; defined and measured trial of labor, VBAC success & VBAC failure, and delivery outcome.
3.3 In the groups being compared (exposed vs non-exposed; with intervention vs without; cases vs controls), are the participants comparable, or do researchers take into account (control for) the difference between these groups?	No	Did not account for or control in analysis for diabetes, or make mention of the choice between family physician and obstetrician.
3.4 Are there complete outcome data (80% or above), and, when applicable, an acceptable response rate (60% or above), or an acceptable follow-up rate for cohort studies (depending on the duration of follow-up)?	Yes	Appears to report all delivery outcomes, complete data.
Overall quality score	☆ ☆ ☆ (75%)	
**Sanchez-Ramos, 1990 (non-concurrent cohort)**		
Are there clear qualitative and quantitative research questions (or objectives), or a clear mixed methods question (or objective)?	Yes	To describe an effort to reduce cesarean sections at a teaching hospital with a guideline-change.
Do the collected data allow address the research question (objective)? E.g. consider whether the follow-up period is long enough for the outcome to occur (for longitudinal studies or study components).	Yes	Delivery outcomes reported, computed annual proportions of primary and repeat cesarean section, trial of labor, VBAC, perinatal & neonatal outcomes
3.1 Are participants (organizations) recruited in a way that minimizes selection bias?	Yes	All eligible women at a teaching hospital were counseled in line with new guideline.

3.2 Are measurements appropriate (clear origin, or validity known, or standard instrument; and absence of contamination between groups when appropriate) regarding the exposure/intervention and outcomes?	Yes	Guideline implementation was July 1, 1987; trial of labor, VBAC, cesarean section rates and other maternal & neonatal outcomes were reported.
3.3 In the groups being compared (exposed vs non-exposed; with intervention vs without; cases vs controls), are the participants comparable, or do researchers take into account (control for) the difference between these groups?	No	No comparison of demographics between groups.
3.4 Are there complete outcome data (80% or above), and, when applicable, an acceptable response rate (60% or above), or an acceptable follow-up rate for cohort studies (depending on the duration of follow-up)?	Yes	Appears to report all delivery outcomes, complete data.
Overall quality score	☆ ☆ ☆ (75%)	
**Santerre, 1996 (non-concurrent cohort)**		
Are there clear qualitative and quantitative research questions (or objectives), or a clear mixed methods question (or objective)?	Yes	To examine if ACOG guideline had an impact on practice of VBACs at typical hospital
Do the collected data allow address the research question (objective)? E.g. consider whether the follow-up period is long enough for the outcome to occur (for longitudinal studies or study components).	Yes	Measured VBAC rates off dataset of 55 hospitals over a 5-year period
3.1 Are participants (organizations) recruited in a way that minimizes selection bias?	Yes	All births for which the mother had a previous cesarean section were studied.
3.2 Are measurements appropriate (clear origin, or validity known, or standard instrument; and absence of contamination between groups when appropriate) regarding the exposure/intervention and outcomes?	Yes	ACOG guideline implementation October 1988, VBAC rate and delivery rates from database used.
3.3 In the groups being compared (exposed vs non-exposed; with intervention vs without; cases vs controls), are the participants comparable, or do researchers take into account (control for) the difference between these groups?	No	No comparison of demographics of groups per year done.
3.4 Are there complete outcome data (80% or above), and, when applicable, an acceptable response rate (60% or above), or an acceptable follow-up rate for cohort studies (depending on the duration of follow-up)?	Yes	Appears to report all delivery outcomes, complete data.
Overall quality score	☆ ☆ ☆ (75%)	
**Studnicki, 1997 (non-concurrent cohort)**		
Are there clear qualitative and quantitative research questions (or objectives), or a clear mixed methods question (or objective)?	Yes	To determine the rate of decrease of cesarean section deliveries after

		a legislatively imposed practice guideline.
Do the collected data allow address the research question (objective)? E.g. consider whether the follow-up period is long enough for the outcome to occur (for longitudinal studies or study components).	Yes	Reported number of cesarean section before and after guideline implementation.
3.1 Are participants (organizations) recruited in a way that minimizes selection bias?	Yes	Discharge data from hospitals studied, included all births from non-federal acute care hospitals.
3.2 Are measurements appropriate (clear origin, or validity known, or standard instrument; and absence of contamination between groups when appropriate) regarding the exposure/intervention and outcomes?	Yes	Measured vaginal delivery & cesarean section for women with previous cesarean (VBAC) and without. Delivery rates taken from discharge data.
3.3 In the groups being compared (exposed vs non-exposed; with intervention vs without; cases vs controls), are the participants comparable, or do researchers take into account (control for) the difference between these groups?	Yes	Basic demographics compared (age, race, pay source); controlled for these characteristics.
3.4 Are there complete outcome data (80% or above), and, when applicable, an acceptable response rate (60% or above), or an acceptable follow-up rate for cohort studies (depending on the duration of follow-up)?	Yes	Appears to report all delivery outcomes, complete data.
Overall quality score	☆ ☆ ☆ ☆ (100%)	
**White, 2016**<br>**(non-concurrent cohort)**		
Are there clear qualitative and quantitative research questions (or objectives), or a clear mixed methods question (or objective)?	Yes	To compare intended and actual VBAC rates before & after midwife led antenatal care.
Do the collected data allow address the research question (objective)? E.g. consider whether the follow-up period is long enough for the outcome to occur (for longitudinal studies or study components).	Yes	Medical records comparing before and after program, delivery rates reported.
3.1 Are participants (organizations) recruited in a way that minimizes selection bias?	Yes	All consecutive women with previous cesarean section in two different cohorts.
3.2 Are measurements appropriate (clear origin, or validity known, or standard instrument; and absence of contamination between groups when appropriate) regarding the exposure/intervention and outcomes?	Yes	Intended and actual mode of birth (VBAC & cesarean) measured.
3.3 In the groups being compared (exposed vs non-exposed; with intervention vs without; cases vs controls), are the participants comparable, or do researchers take into account (control for) the difference between these groups?	Yes	Baseline demographics were similar.

3.4 Are there complete outcome data (80% or above), and, when applicable, an acceptable response rate (60% or above), or an acceptable follow-up rate for cohort studies (depending on the duration of follow-up)?	Yes	Records for 15/424 (4%) unavailable, complete outcome data reported.
Overall quality score	☆ ☆ ☆ ☆ (100%)	
**Wong, 2014 (prospective cohort)**		
Are there clear qualitative and quantitative research questions (or objectives), or a clear mixed methods question (or objective)?	Yes	To evaluate how obstetrician-led cesarean section education & antenatal sessions influences mode of delivery for women who have a previous cesarean.
Do the collected data allow address the research question (objective)? E.g. consider whether the follow-up period is long enough for the outcome to occur (for longitudinal studies or study components).	Yes	VBAC rates measured.
3.1 Are participants (organizations) recruited in a way that minimizes selection bias?	Yes	All women with previous cesarean section in calendar year within one hospital, no contraindications, were invited to session.
3.2 Are measurements appropriate (clear origin, or validity known, or standard instrument; and absence of contamination between groups when appropriate) regarding the exposure/intervention and outcomes?	Yes	VBAC rates measured, and elective cesarean.
3.3 In the groups being compared (exposed vs non-exposed; with intervention vs without; cases vs controls), are the participants comparable, or do researchers take into account (control for) the difference between these groups?	No	Did not report or compare demographics between groups.
3.4 Are there complete outcome data (80% or above), and, when applicable, an acceptable response rate (60% or above), or an acceptable follow-up rate for cohort studies (depending on the duration of follow-up)?	Unsure	Those who did not attend intervention were slotted as comparator group -78/266 eligible (29%); 188/266 (71%) attended the group.
Overall quality score	☆ ☆ (50%)	
**Yee, 2017 (retrospective cohort)**		
Are there clear qualitative and quantitative research questions (or objectives), or a clear mixed methods question (or objective)?	Yes	To investigate the relationship between obstetrician's call schedule and obstetric outcomes
Do the collected data allow address the research question (objective)? E.g. consider whether the follow-up period is long enough for the outcome to occur (for longitudinal studies or study components).	Yes	Medical records of women with previous cesarean section and no vaginal delivery; reported VBAC rates/ trial of labor assessed.
3.1 Are participants (organizations) recruited in a way that minimizes selection bias?	Yes	All deliveries of women with prior cesarean section without vaginal

		delivery, abstracted from medical records.
3.2 Are measurements appropriate (clear origin, or validity known, or standard instrument; and absence of contamination between groups when appropriate) regarding the exposure/intervention and outcomes?	Yes	Clear definitions for call schedules. Birth rates measured with TOLAC, VBAC attempt & success, maternal and neonatal outcomes.
3.3 In the groups being compared (exposed vs non-exposed; with intervention vs without; cases vs controls), are the participants comparable, or do researchers take into account (control for) the difference between these groups?	Yes	Patient characteristics stratified by physician call type; controlled for patient characteristics significantly associated with call type in regression analysis.
3.4 Are there complete outcome data (80% or above), and, when applicable, an acceptable response rate (60% or above), or an acceptable follow-up rate for cohort studies (depending on the duration of follow-up)?	Yes	Appears to report all delivery outcomes, complete data.
Overall quality score	☆ ☆ ☆ ☆ (100%)	
**Zhang, 2016**<br>**(RCT)**		
Are there clear qualitative and quantitative research questions (or objectives), or a clear mixed methods question (or objective)?	Yes	To determine if midwifery care has more benefits than standard maternity care in VBAC.
Do the collected data allow address the research question (objective)? E.g. consider whether the follow-up period is long enough for the outcome to occur (for longitudinal studies or study components).	Yes	All births reported, rates of VBAC, fetal distress and other maternal characteristics.
2.1 Is there a clear description of the randomization (or an appropriate sequence generation)?	Unsure	No description of randomization.
2.2 Is there a clear description of the allocation concealment (or blinding when applicable)?	Unsure	No description of allocation concealment.
2.3 Are there complete outcome data (80% or above)?	Yes	All participants randomized are reported on.
2.4 Is there low withdrawal/drop-out (below 20%)?	Unsure	Did not report on withdrawals or dropouts.
Overall quality score	☆(25%)	
**Zweifler, 2016**<br>**(non-concurrent cohort)**		
Are there clear qualitative and quantitative research questions (or objectives), or a clear mixed methods question (or objective)?	Yes	To assess VBAC trends before and after guideline revision and compare neonatal and maternal morbidity.
Do the collected data allow address the research question (objective)? E.g. consider whether the follow-up period is long enough for the outcome to occur (for longitudinal studies or study components).	Yes	Measured birth rates and birth statistics.
3.1 Are participants (organizations) recruited in a way that minimizes selection bias?	Yes	Birth data and maternal demographics obtained from state

		department records for all women with previous cesarean section.
3.2 Are measurements appropriate (clear origin, or validity known, or standard instrument; and absence of contamination between groups when appropriate) regarding the exposure/intervention and outcomes?	Yes	ACOG guideline revision, VBAC birth rates (success or failure), cesarean section rates, and maternal & neonatal outcomes measured.
3.3 In the groups being compared (exposed vs non-exposed; with intervention vs without; cases vs controls), are the participants comparable, or do researchers take into account (control for) the difference between these groups?	Yes	Compared demographics for years before and after guideline revision.
3.4 Are there complete outcome data (80% or above), and, when applicable, an acceptable response rate (60% or above), or an acceptable follow-up rate for cohort studies (depending on the duration of follow-up)?	Yes	Appears to report all delivery outcomes, complete data.
Overall quality score	☆ ☆ ☆ ☆ (100%)	

VBAC: vaginal birth after cesarean; TOLAC: trial of labor after cesarean; ACOG: The American College of Obstetricians and Gynecologists; RCT: randomized controlled trial; h: hour; vs. versus; OBGYN: Obstetrician-Gynecologist

*Assessed using the Mixed Methods Appraisal Tool, Version 2011
